# Robust expression of LINE-1 retrotransposon encoded proteins in oral squamous cell carcinoma

**DOI:** 10.1186/s12885-021-08174-z

**Published:** 2021-05-27

**Authors:** Koel Mukherjee, Debpali Sur, Abhijeet Singh, Sandhya Rai, Neeladrisingha Das, Rakshanya Sekar, Srinu Narindi, Vandana Kumar Dhingra, Bhinyaram Jat, K. V. Vinu Balraam, Satya Prakash Agarwal, Prabhat Kumar Mandal

**Affiliations:** 1 Department of Biotechnology, IIT Roorkee, Roorkee, Uttarakhand India; 2 Department of Head-Neck Surgery and Oncology, AIIMS Rishikesh, Rishikesh, Uttarakhand India; 3 School of Biosciences and Technology, Vellore Institute of Technology, Vellore, Tamil Nadu India; 4 Military Hospital, Roorkee, Uttarakhand India

**Keywords:** L1 retrotransposon, Cancer and L1 retrotransposon, L1ORF2p antibody, L1ORF1p antibody, OSCC and L1 retrotransposon

## Abstract

**Background:**

Oral Squamous Cell Carcinoma (OSCC) results from a series of genetic alteration in squamous cells. This particular type of cancer considers one of the most aggressive malignancies to control because of its frequent local invasions to the regional lymph node. Although several biomarkers have been reported, the key marker used to predict the behavior of the disease is largely unknown. Here we report Long INterpersed Element-1 (LINE1 or L1) retrotransposon activity in post-operative oral cancer samples. L1 is the only active retrotransposon occupying around 17% of the human genome with an estimated 500,000 copies. An active L1 encodes two proteins (L1ORF1p and L1ORF2p); both of which are critical in the process of retrotransposition. Several studies report that the L1 retrotransposon is highly active in many cancers. L1 activity is generally determined by assaying L1ORF1p because of its high expression and availability of the antibody. However, due to its lower expression and unavailability of a robust antibody, detection of L1ORF2p has been limited. L1ORF2p is the crucial protein in the process of retrotransposition as it provides endonuclease and reverse transcriptase (RT) activity.

**Methods:**

Immunohistochemistry and Western blotting were performed on the post-operative oral cancer samples and murine tissues.

**Results:**

Using in house novel antibodies against both the L1 proteins (L1ORF1p and L1ORF2p), we found L1 retrotransposon is extremely active in post-operative oral cancer tissues. Here, we report a novel human L1ORF2p antibody generated using an 80-amino-acid stretch from the RT domain, which is highly conserved among different species. The antibody detects significant L1ORF2p expression in human oral squamous cell carcinoma (OSCC) samples and murine germ tissues.

**Conclusions:**

We report exceptionally high L1ORF1p and L1ORF2p expression in post-operative oral cancer samples. The novel L1ORF2p antibody reported in this study will serve as a useful tool to understand why L1 activity is deregulated in OSCC and how it contributes to the progression of this particular cancer. Cross-species reactivity of L1ORF2p antibody due to the conserved epitope will be useful to study the retrotransposon biology in mice and rat germ tissues.

**Supplementary Information:**

The online version contains supplementary material available at 10.1186/s12885-021-08174-z.

## Introduction

Retrotransposons (jumping genes) are sequences which move from one place in the genome to another using RNA as an intermediate, and are responsible for individual cases of many genetic disorder due to disruption of essential genes [1–3]. The human genome harbors a retrotransposon named Long INterspersed Element (LINE-1 or L1) which is highly active as evidenced by 500,000 copies occupying around 17% of the human genome [4, 5]. In addition to copying itself to new genomic locations, L1 activity is also responsible for the formation of over one million other retrotransposon insertions (e.g., Alu and SVA elements) and several thousand processed pseudogenes in the human genome [6–9].

Although highly abundant, only a subset of L1s (~ 80–100 copies) are actively retrotransposing in any given human [10]. A retrotransposition-competent L1(RC-L1) is 6 kb in length with the following features: a 5′-UTR (~ 900 bp) with an internal promoter, two non-overlapping open reading frames (ORFs designated L1ORF1p and L1ORF2p) separated by a 63 bp spacer sequence, and a ~ 200 bp 3′-UTR ends with a poly (A) sequences with variable length (10–400 bp) [11–13]. The element is surrounded by target site duplications (TSDs) that vary both in size and sequence [2]. Human L1 ORF1 encodes a 40 kDa protein (338 amino acids in length) termed ORF1p comprised of three distinct domains; Coiled Coil (CC) (amino acids 52–153), RNA Recognition Motif (RRM) (amino acids 157–252), and Carboxy Terminal Domain (CTD) (amino acids 264–323) [14, 15]. In-vitro studies have demonstrated that human ORF1p is a non-specific single-stranded nucleic acid-binding protein with nucleic acid chaperone activity [14–16]. Human ORF2 encodes a 150 kDa protein with reverse transcriptase (RT) [17] and endonuclease (EN) [18] activities. Functional studies have revealed that both ORF1p and ORF2p are critical for retrotransposition of their encoding RNA [13].

Due to their potential to function as insertional mutagens, L1s are generally silenced in somatic cells through epigenetic and post-transcriptional mechanisms. These include CpG methylation of the L1 promoter; small RNA induced silencing, cellular host factor-mediated retrotransposition inhibition and others [19, 20]. However, recent transgenic animal models and deep-sequencing studies revealed that a subset of L1s escape repression and show high activity in germ cells, early stages of development, certain parts of the brain and in cancers [21–27]. The whole-genome (WGS) and targeted sequencing of tumour samples showed high rates of L1 retrotransposition in many cancers, particularly those of epithelial cell origin [23]. Notably, in some cancers (e.g., colorectal cancer), the somatic L1 insertion frequency is striking with more than 100 retrotransposition events detected in one tumor [23], while in other tumors, retrotransposition is not found. Immunohistochemical analysis using human L1 ORF1p antibody showed that nearly half of human cancers are immune-reactive with LINE-1 ORF1p [25]. Recently, in a pilot study with a small number of samples, we have demonstrated significant expression of L1ORF1p in post-operative oral cancer, a cancer which is extremely common in India due to excessive use of tobacco [27]. The L1ORF2p is the central protein required in the process of retrotransposition and is very difficult to detect due to its low expression and lack of a specific antibody [13, 28–31]. Although our understanding of L1 activity in cancers has increased dramatically over the past five years, very few studies have been conducted to determine the expression of L1 ORF2p in cancers due to lack of a proper ORF2p antibody [32–35]. A few groups have reported an L1ORF2p antibody [32–35]; however, questions have been raised regarding the specificity and sensitivity of those antibodies.

Here we report the successful development of a human L1ORF2p antibody using an 80 amino acid stretch from the reverse transcriptase domain of L1ORF2p. Alignment of this human stretch with mouse and rat L1ORF2p sequences showed that the stretch is highly conserved among all three species [36]. The developed L1ORF2p antibody detects significant ORF2p expression in murine testis and ovary and in post-operative oral cancer samples. Until now, no study has reported L1ORF2p expression in oral cancer. We have screened 39 post-operative oral cancer samples and found that more than 50 % of samples expressed L1ORF2p. Overall, we report a novel ORF2p antibody which detects L1 activity in germ tissues and post-operative oral cancer samples.

## Materials methods

### Cloning of human hL1RT_EH_ fragment

**Fig. 1:**
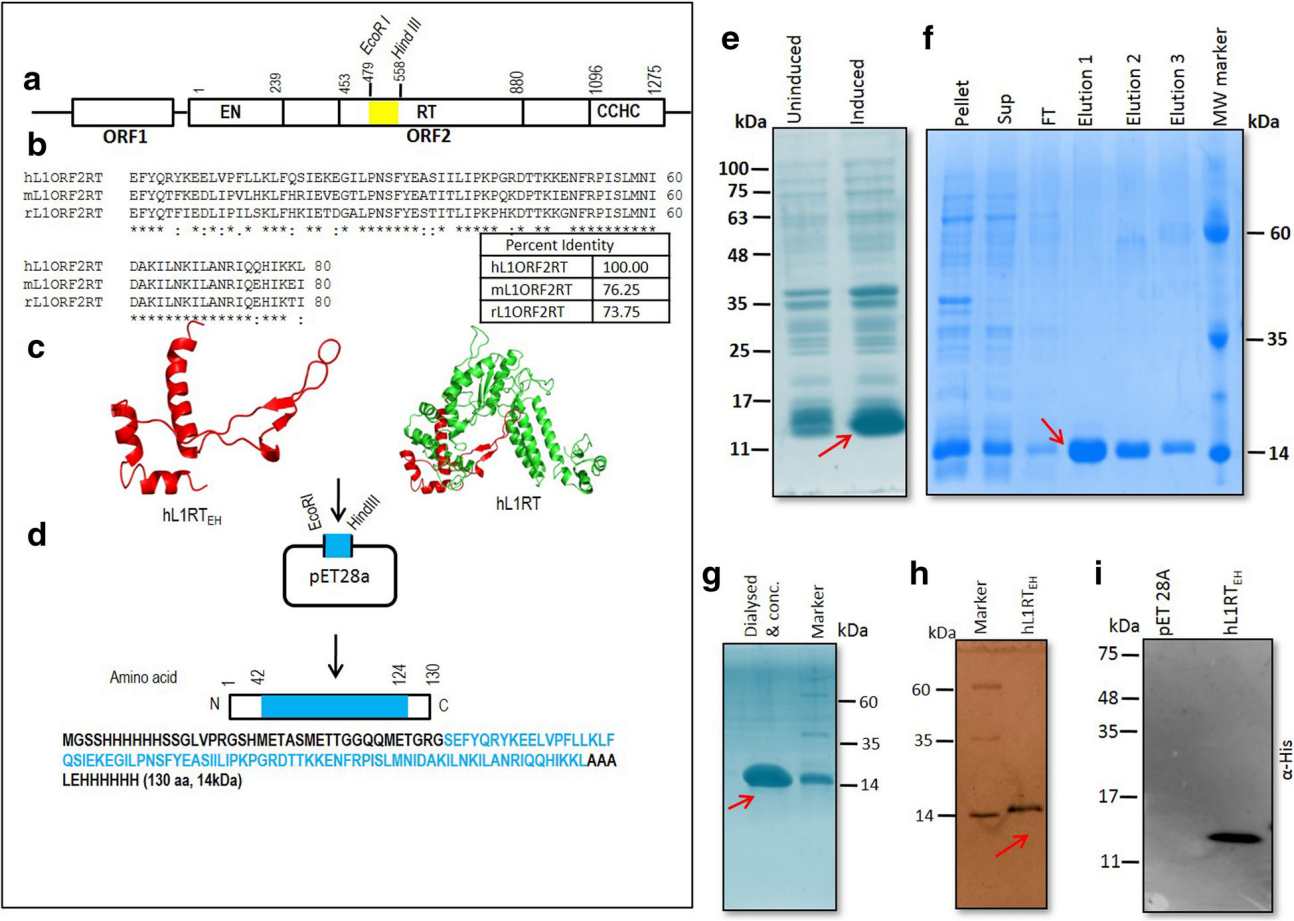
Generation of the antigen for the production of Human L1 ORF2 specific antibody: **a** Schematic diagram of full length active human L1 retrotransposon with two encoded proteins (L1ORF1p and L1ORF2p). L1ORF2p (1275 amino acids in length with predicted MW 150 kDa) has three partially characterized domains: endonuclease (EN) (AA:1–239), reverse transcriptase (RT) (AA: 453–883) and a cysteine-histidine-rich domain (CCHC) (AA: 1096–1275). The *EcoRI-HindIII* restriction fragment (AA: 479–558 fragment name hL1RT_EH_) from the RT region was used as an antigen to make antibody against hL1ORF2p (shaded by yellow). **b** Alignment of the selected eighty amino acids stretches of the RT domain (hL1RT_EH_) among human, mouse and rat L1. The selected RT stretch showed 76.25 and 73.75% identity at the protein level with the same stretch present in mice and rat L1ORF2, respectively. **c** Predicted structure of hL1RT_EH_ fragment and complete human RT domain (generated using PyMOL) [38]. **d** Sub-cloning scheme of human hL1RT_EH_ fragment in pET-28a bacterial expression vector. Bacterial expressed hL1RT_EH_ fragment encodes 130 amino acid polypeptides (from N terminal to C terminal AA: 1–42 vector, 43–124 RT fragment, and 125–130 vector) with predicted MW around 14 kDa. **e** Whole-cell lysate SDS-PAGE of *E.coli* expressed pET-hL1RT_EH_. Induced protein with a molecular weight around 14 kDa is shown by the arrow. **f** Purification of hL1RT_EH_ from inclusion bodies by dissolving the pellet fraction in 8 M urea buffer (details in materials and methods). The purified protein in elution 1, 2 and 3 is shown by an arrow. **g** Dialysed and concentrated hL1RT_EH_ fragment protein (antigen) injected to mice for antibody generation. **h** Silver staining of purified hL1RT_EH_ (antigen) show the purity of antigen used to generate the antibody. **i** Western blotting of hL1RT_EH_ using an anti-His antibody. MW: Molecular Weight, FT: Flow-through

The human L1 ORF2pRT domain fragment (234 bp *EcoRI -HindIII* fragment, Nucleotide position in L1ORF2 1435–1674, amino acid position in L1ORF2 479–558, L1RP accession number AF148856) [37] was cloned in pET28a to make a pEThL1RT_EH_ clone. The cloning strategy is provided in Fig. 1 and Supplementary Fig. 1.

### Expression and purification of human hL1RT_EH_ domain fragment protein

The pEThL1RT_EH_ clone was transformed to *E.coli* (strain BL21) expression cells, plated to Kanamycin containing agar plates and incubated at 37 °C overnight. A single colony was incubated overnight in 10 ml LB media with an appropriate antibiotic (Kanamycin 25 μg/ml) to obtain primary culture. One percent of the primary culture was added to 100 ml LB media and the culture was grown at 37 °C till OD_600_ 0.4 before the addition of 0.4 mM isopropyl β-D-thiogalactopyranoside (IPTG) for the induction of hL1RT_EH_ at 37 °C for another 3 h. After the induction, cells were harvested, resuspended in lysis buffer [50 mM Tris-Cl (pH -8.0), 150 mM NaCl, 10 mM imidazole, 1 mM PMSF]. The cells were then lysed by three cycles of freeze-thawing followed by sonication on ice. The lysate was centrifuged at 12000 x g for 30 min at 4 °C. As the hL1RT_EH_ formed inclusion bodies, the supernatant was discarded, and the pellet was dissolved in buffer A (50 mM Tris-Cl pH 8.0, 150 mM NaCl, 10 mM imidazole, 8 M Urea) followed by centrifugation at 12000 x g for 20 min at 4 °C. The supernatant was collected to a separate tube, and the pellet was discarded. Next, the supernatant was incubated with pre-equilibrated nickel agarose resin (Qiagen) (100 ul packed resin per 50 ml culture) for one hour at 4 °C with continuous shaking. The resin was washed with 5 ml wash buffer (50 mM imidazole). The protein was finally eluted in elution buffer (50 mM Tris-Cl pH 8.0, 150 mM KCl, 300 mM imidazole, 6 M Urea). The urea from the protein solution was removed by serial dialysis to avoid precipitation of protein, i.e. 8 M Urea➔6 M Urea➔ 4 M Urea➔ 2 M Urea➔ 1 M Urea➔ 0.8 M Urea➔ 0.6 M➔0.4 M➔0.2 M➔0 M Urea. After dialysis, the protein was concentrated using a centrifugal filter unit (MWCO 3000 Da) and stored at − 20 °C after a flash freeze. Purified protein fragment with a molecular mass of approximately 14 kDa (vector sequence plus eighty amino acid RT fragment, details in Fig. 1 and supplementary text) was used to immunize mice (Swiss Albino).

### Generation of polyclonal antibody against hL1RT_EH_ in mice

The purified hL1RT_EH_ protein was used as an antigen for raising its antibody in swiss albino mouse. The 80 days immunization protocol followed is presented below in details:

**Table:** 

PROCEDURE	PROTOCOL	DESCRIPTION
Control serum collection	Day 0	Pre-immune bleed
Primary injection	Day 1	Immunize with 100 μg antigen in complete Freud’s adjuvant
1st Booster	Day 21	Boost with 50 μg antigen in incomplete Freud’s adjuvant, 1st bleed collection
2nd Booster	Day 42	Boost with 50 μg antigen in incomplete Freud’s adjuvant, 2nd bleed collection
3rd Booster	Day 62	Boost with 50 μg antigen in incomplete Freud’s adjuvant, 3rd bleed collection
Sacrifice	Day 80	Terminal Bleed collection and termination of animals, 4th bleed collection

Serum was isolated from blood collected in each step by incubating the blood for 1 h, in 37 °C water bath followed by incubating a 4 °C over-night. Next day the sample was centrifuged (12,000×g, 10 min, 4 °C), and the serum was collected.

### Purification of IgG fraction of anti-hL1RT_EH_ from whole immunized serum

Around 500 μl of total serum obtained from hL1RT_EH_ fragment immunized mice was mixed in 1:1 ratio with binding buffer (0.02 M sodium phosphate, pH 7.0). Around 100 μl packed protein G agarose (GE Healthcare, cat no: 1812776) slurry (stored in 20% ethanol) was taken and equilibrated with binding buffer. Next, serum and binding buffer mix (ratio1:1, Volume 1000 μl) was added to the beads and incubated for 1 h at 4 °C (in rocker) for effective binding of IgG with protein G agarose. After incubation, centrifuged the tube at 2000 xg for one min at 4 °C, and incubated the tube on ice for one minute to settle the beads. The flow-through was carefully removed and stored for further analysis. Next, the beads were washed with 5 × 1 ml wash buffer (0.02 M sodium phosphate, pH 7.0). Finally, the IgG fraction was eluted in 100 μl elution buffer (0.1 M Glycine, pH 2.5) by centrifugation at 2000 x g for 2 min at 4 °C. Immediately the eluate was neutralized with 23 μl of neutralization buffer (1 M Tris-Cl, pH -9.0). The antibody concentraion was carried out using Bradford assay, and the quality of the purified antibody was checked in 10% SDS-PAGE gel. The affinity-purified antibody was checked and confirmed by performing immunoblotting.

### Animals

The Swiss albino mice and Sprague Dawley rats were procured from the animal facility of National Institute of Pharmaceutical Education and Research (NIPER), Chandigarh, India were housed in the animal facility at Indian Institute of Technology Roorkee, India. All the experiments were carried out as per indicated guidelines of the Institute Animal Ethics Committee (IAEC) under Protocol no: BT/IAEC/2018/11 and BT/IAEC/2018/12. All the animal experimental protocols were approved by the Committee for the Purpose of Control and Supervision of Experiments on Animals (CPCSEA). The complete study was carried out in compliance with the ARRIVE guidelines.

### Cell culture

HEK293T, (human embryonic kidney) and HeLa (cervical carcinoma) cells obtained from National Centre for Cell Science (NCCS), Pune, India were maintained in a CO_2_ incubator at 37 °C and 5% CO_2_ concentration in high glucose Dulbecco’s modified Eagle medium (DMEM) with L– glutamine (Gibco, cat no: 12100046) supplemented with 10% fetal bovine Calf serum (Gibco cat no: 26140087) and 100 U/ml penicillin-streptomycin (Gibco, cat no: 10378–016).

### Plasmids and cell transfection

Around 2 × 10^5^ HEK293T were seeded on a 35 mm tissue culture plate. After 8–10 h incubation, 1 μg of L1-EGFP construct was transfected into cells using Fugene 6 Transfection Reagent (Promega, cat no: E2691). One day after transfection the old media was replaced with new media; 96 h after post-transfection EGFP positive cells were checked under the microscope to proceed for immune-blotting.

### Cells and tissue lysate preparation and immunoblotting

Whole-cell lysates from cell lines were prepared using NP40 lysis buffer [20 mM Tris-Cl pH 7.8‚ 137 mM NaCl and 1% NP-40 supplemented with 1X protease inhibitor cocktail (Roche, P8340)]. The tissue lysate was prepared from mouse, rat and OSCC tissues using RIPA buffer [50 mM Tris-Cl pH 8.0, 150 mM NaCl, 0.5% sodium deoxycholate, 0.1% SDS and 1% NP-40 supplemented with 1X protease inhibitor cocktail]. The lysate was centrifuged at 10,000 g for 10 min at 4 °C, and the supernatant was transferred to a new 1.5 ml tube and stored at − 70 °C until further use. Around 40 μg of whole-cell lysate was resolved in 10% SDS-PAGE gel (Mini protein Tetra cell Bio-Rad) and blotted to nitrocellulose membrane (Bio-Rad) by applying 100 V for 75 min using Bio-Rad mini trans blot electrophoretic transfer cell (transfer buffer composition:25 mM Tris-Cl pH 7.6, 192 mM Glycine, 0.1% SDS, 20% Methanol). The membrane was probed with Primary: polyclonal mouse anti-hL1RT_EH_ (1:5000), anti-hL1-ORF1 (RRM) (1:33000) [26], anti-GAPDH (1:2000) (Santacruz Biotechnology, cat no: sc166545). The next day, the membrane was washed three times for 15 min with 1x TBST and incubated with secondary antibody [α-rabbit HRP 1: 20000 (Jacksons Immuno Research Laboratories, USA, cat no: 111–035-003) and α-mouse HRP 1:20000 (Jacksons Immuno Research Laboratories, USA, cat no: 115–035-174). Western blots were developed using ECL western blotting detection reagent (Biorad) as per the manufacturer’s instructions. The bands were detected by exposing the blot on X-ray film (Fujifilm).

### Sample collection of cancer tissue specimens

Post-operative cancer tissues were collected from the surgical oncology department, AIIMS Rishikesh, following proper written informed consent from the patient and their immediate family members as per institute guidelines. The patient and/or the family members understood and agreed that a small portion (2–5 g) of operated cancer tissue will be used for research purpose to understand the biology of oral cancer and its treatment. Following initial collection, samples were stored in RNA later solution (Qiagen) at − 20 °C and used for lysate preparation. The tissue samples stored in 10% NBF solution were subsequently used for the generation of formalin-fixed paraffin embedded blocks (FFPE). All the experiments were conducted in accordance with ethical principles embodied in the declaration of tissue request and material transfer agreement between AIIMS Rishikesh and IIT Roorkee. The approval from the institutional ethics committee of All India Institute of Medical Sciences, Rishikesh (Reg No: ECR/736/Inst/UK/2015/RR-18) has been obtained specifically for the work with human samples (Letter No: AIIMS/IEC/20/395). All the investigations in this study strictly followed the rules set by the Declaration of Helsinki.

### Hematoxylin-eosin staining

FFPE tissue sections of 4 μm were deparaffinised using xylene followed by rehydration in descending grade of ethanol solutions. Then the slides were stained with Mayer’s hematoxylin (Himedia) for three minutes, washed in tap water and counterstained with eosin Y solution (Himedia) for one minute. The slides were further dehydrated with ascending grade of ethanol and xylene solutions, and finally, the tissue sections were examined under an upright light microscope (Leica Microsystems) after mounting with DPX mounting media (Himedia).

### Immuno histochemistry

Formalin-fixed paraffin-embedded (FFPE) tissues were sectioned to four micron thickness on coated glass slides. They were deparaffinized, and rehydrated in descending grades of ethanol solutions before proceeding for antigen retrieval. The antigen retrieval was performed in a common household vegetable steamer (pressure cooker) using Tris-EDTA antigen retrieval buffer (10 mM Tris base, 1 mM EDTA solution, 0.05% Tween 20, pH -9.0). Next, the slides were washed 2 X 5 min each in TBST buffer (1X TBS containing 0.025% Triton-X100) and then blocked in blocking solution (3% BSA in 1XTBST) for 1 h at room temperature. For ORF1p staining, slides were incubated with polyclonal rabbit α-ORF1p (RRM) antibody (1:500 diluted in blocking solution), and for ORF2p we probed the slides with polyclonal mouse anti-hL1RT_EH_ (1:200 diluted in blocking reagent) and kept at one hour at room temperature or 4 °C overnight in a humid chamber. The next day, slides were washed three times for ten minutes with 1XTBST. Endogenous peroxidase activity was quenched by treating the slides with 0.3% hydrogen peroxide. Slides were then incubated with secondary antibody [α-rabbit HRP 1:500 (Jacksons Immuno Research, cat no: 111–035-003) and α-mouse HRP 1: 500 (Jacksons Immuno Research, cat no: 115–035-174)] for an hour at room temperature. The slides were washed again three times for ten min with 1X TBST at room temperature with gentle agitation. All the signals were visualized by adding 3–3′- Diaminobenzidine tetrahydrochloride (DAB substrate) solution (Roche) to the slides and counterstained with haematoxylin (Himedia). Following counterstaining, the slides are dehydrated with ascending order of ethanol and mounted with DPX mounting media (Himedia). Anti-GAPDH (1:250) (Santacruz Biotechnology, cat no: sc166545) was used as housekeeping control, PanCK (pre-diluted from PathnSitu biotechnology India, cat no: R05138UA) and Ki67 (pre-diluted from PathnSitu biotechnology India, cat no: R05210MA) were used as squamous cell carcinoma marker and cell proliferation marker respectively. p53 (pre-diluted from PathnSitu biotechnology India, cat no: R06101LA) expression was also examined. Images were captured using an upright light microscope (Leica Microsystems) equipped with a camera. All the microscopic pictures were taken at 40X magnification.

## Results

### Generation of a novel polyclonal antibody against L1-ORF2p

Human L1 ORF2p protein is 1275 amino acid residues in length (L1RP, Accession number: AF148856.1) with a predicted molecular weight (MW) of~ 150 kDa. It has three partially characterized domains, which are from N-C terminal: endonuclease (EN) (AA: 1–239), reverse transcriptase (RT) (AA: 453–883) and a cysteine/ histidine-rich domain (CCHC) (AA: 1096–1275) [13] (Fig. 1a). The L1ORF2p is the central protein required in the process of retrotransposition with demonstrated RT and EN activities [17, 18]. Due to its very low expression, the detection of ORF2p is extremely challenging [28–31]. Deciphering the expression level of human L1-ORF2p across various somatic, germ line and cancerous tissues could lead to a better understanding of L1 biology.

In this study, we have generated a highly specific polyclonal antibody against human L1-ORF2p. Through bioinformatics analysis, we identified an 80 amino acid stretch present in the RT domain [240 bp *EcoRI- HindIII* fragment of L1ORF2, Nucleotide position in L1ORF2 1435–1674, amino acid position in L1ORF2 479–558, (L1RP accession number AF148856)], to be evolutionarily conserved across various species (Fig. 1a and b). Multiple sequence alignment results showed that the selected RT stretch is 76.25 and 73.75% identical at the protein level with mouse and rat L1 RT, respectively (Fig. 1b and supplementary text). The *in slico* structural study showed that the selected stretch is open and situated outside of the folded RT structure; thus, it can be a suitable epitope for antibody generation (Fig. 1c).

As the selected region is flanked by natural restriction enzymes *EcoRI* and *HindIII* in L1RP [37], the JCC5 clone (L1RP cloned in pBS) was digested with *EcoRI* and *HindIII* to get the desired fragment (234 nucleotides) and then sub-cloned into a bacterial expression vector pET-28A (Fig. 1a, Supplementary Fig. 1A). The resultant clone (named hL1RT_EH_), if expressed in a bacterial cell, will produce a 130 amino acid polypeptide (from N terminal to C terminal AA: 1–42 vector, 43–124 RT fragment, and 125–130 vector) with MW around 14 kDa (Fig. 1d). The N-terminal 6X His-tagged hL1RT_EH _was expressed in *E. coli* expression cells, and a distinct band of around 14 kDa was detected in the pellet fraction, suggesting that the induced protein formed inclusion bodies (Fig. 1e and f). The protein was then purified from inclusion bodies by dissolving the pellet in urea, followed by Ni-agarose chromatography (Fig. 1f). Analysis of the purified hL1RTR_EH _fragment by SDS-PAGE followed by Coomassie and silver staining revealed a distinct band at around 14 kDa with ~ 100% purity (Fig. 1g and h). The purified hL1RT_EH_ was also confirmed by Western blotting using an anti-His antibody before injecting to mouse (swiss albino) for antibody production (Fig. 1i). The mouse pre-immune serum was checked by western blotting, and no cross-reactivity was detected (Supplementary Figure1B).

### Characterization of anti-hL1ORF2 by immunoblotting

**Fig. 2:**
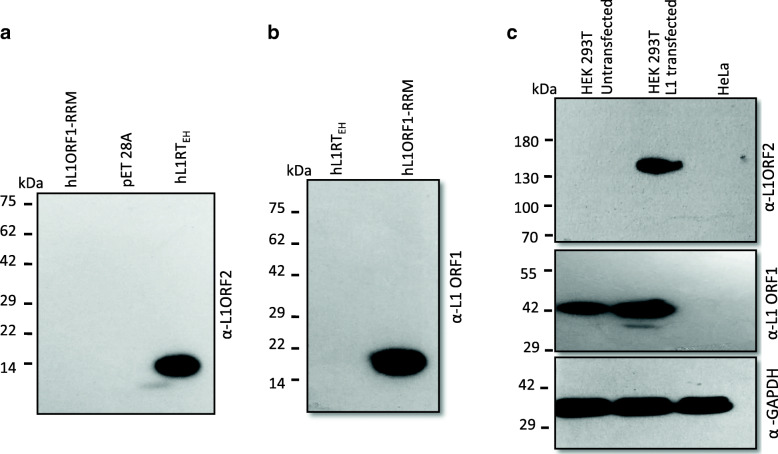
Characterization of human L1 ORF2p antibody (anti-hL1ORF2): **a**) Immunoblot analysis with anti-hL1ORF2RT_EH_ on induced bacterial lysate obtained from and pEThL1ORF1_RRM_, pET28A and pET-hL1RT_EH_. Human L1ORF1p RRM antigen used to make anti-hL1ORF1p didn’t show any cross-reaction with anti-hL1ORF2p [26, 27] (**b**) Immunoblot analysis with anti-hL1ORF1_RRM_ on total bacterial lysate obtained from pET-hL1RT_EH_, and pEThL1ORF1_RRM_. No cross-reaction of anti-hL1ORF1with hL1RT_EH_ fragment used as antigen to make ORF2p antibody [26]. **c** Detection of L1ORF2p (exogenous), L1ORF1p (endogenous and exogenous) and GAPDH (endogenous) [26, 27] by immunoblotting in HEK 293T cells after transfecting full-length L1 construct (pL1_RP_EGFP) [39]. All the original immuno-blots are shown in supplementary file

The mice anti-serum raised against hL1RT fragment was checked by Western blotting using IPTG-induced total lysate obtained from bacterial expression cells containing the pET-hL1RT_EH _clone (Fig. 2a). A single band at around 14 kDa was detected in that lane, and the control lanes lacked any signals (total lysate from induced empty vector pET28a and pET-hRRM clone; ORF1p RRM domain cloned in pET30b) [26] (Fig. 2a). This result suggests that the selected ORF2p RT fragment (amino acids 479–558 of L1ORF2p) is immunogenic in mice and does not cross-react with human L1ORF1p protein (Fig. 2a). Recently, we have reported a novel hL1ORF1 antibody where we use the RRM domain of human L1ORF1p as an antigen [26, 27]. In order to check whether anti-hL1ORF1 cross-reacts with the hL1ORF2pRT_EH_ fragment, a Western blot was performed with bacterial over-expressed pET-hRT_EH_, and no band was detected, suggesting that anti-hL1ORF1p does not cross-react with hORF2RT_EH_ peptide (Fig. 2b).

Next, to test the anti-hRT_EH_ antibody for its ability to detect native ORF2p in situ, we transfected episomal retrotransposon reporter plasmid pL1_RP_EGFP (full-length disease-causing L1 with retrotransposon indicator cassette cloned in a pCEP4 vector) [39] into HEK293T cells. We performed Western blotting using total lysate 96 h post-transfection. The data showed a distinct band at around 150 kDa, corresponding to the proposed MW of ORF2p in the transfected lane, whereas no signal was detected in lysate obtained from untransfected cells (Fig. 2c and Supplementary Fig. 2A). The same samples were checked for the presence of hORF1p, and the Western blot showed the presence of ORF1p in both transfected and untransfected cells (endogenous ORF1p) (Fig. 2c). Previous studies showed a significant amount of endogenous ORF1p expression in HEK293T cells [27]. GAPDH was used as a loading control (Fig. 2c). From the above result, it is evident that the generated antibody is highly specific and detects L1ORF2p as a discrete single band.

Next, to check the sensitivity of anti-L1ORF2p antibody, bacterial over-expressed pET-hORF2RT_EH_and pL1_RP_EGFP [39] transfected HEK293T total lysate were separated in SDS-PAGE gel in increasing concentration, and Western blotting was performed. The data showed that the anti-ORF2p could detect as little as 10 ng and 20 μg in over-expressed bacterial and L1 transfected HEK293T total lysate, respectively (Supplementary Fig. 2B).

### Detection of endogenous L1ORF2p and L1ORF1p in different somatic and germ line tissues

**Fig. 3:**
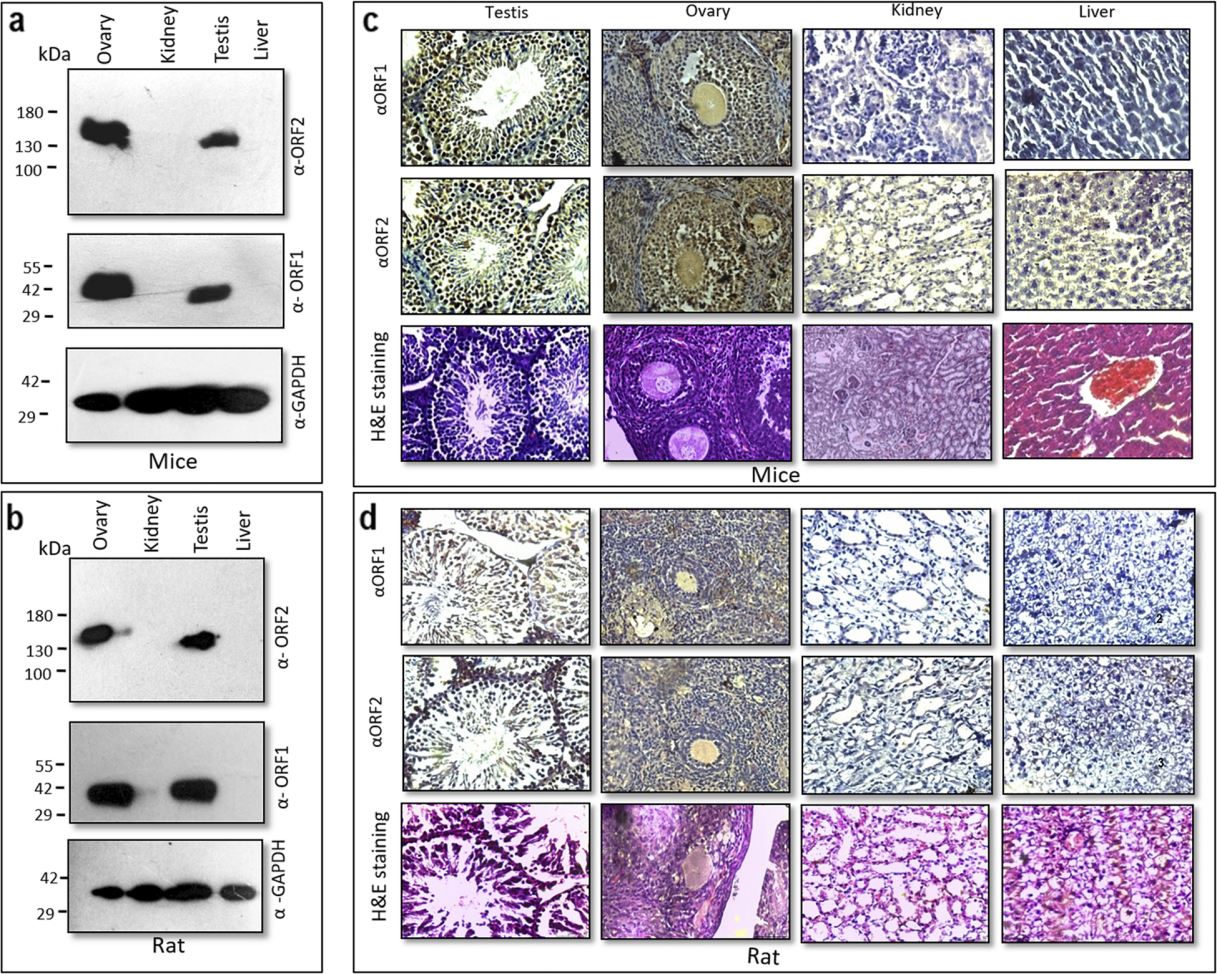
Detection of L1ORF1p and L1ORF2p in mouse and rat tissues: **a** Immunoblot analysis of somatic and germline tissues of the mouse (testis, ovary, liver and kidney) with anti-L1ORF2 (panel 1), anti-L1ORF1 (panel 2), and anti-GAPDH (panel 3) as loading control. **b** Immunoblot analysis of somatic and germline tissues of rat (testis, ovary, liver and kidney) with anti- L1ORF2 (panel 1), anti-L1ORF1 (panel 2), and GAPDH (panel 3) as loading control. Original Western blots are attached in supplementary file. **c** Immunohistochemical analysis of somatic and germline tissues of the mouse (testis, ovary, liver and kidney) with anti- L1ORF1 (panel 2) and anti- L1ORF2 (panel 3). Tissue sections stained with hematoxylin-eosin are shown in panel 1. **d** Immunohistochemical analysis of somatic and germline tissues of rat (testis, ovary, liver and kidney) with anti-L1ORF1 (panel 2) and anti-L1ORF2 (panel 3). Hematoxylin-eosin stained samples are shown in panel 1

It is well known that L1 retrotransposon activity is generally attenuated in somatic cells/tissues, while numerous reports indicate high L1 activity in germ line cells and tissues [22, 29, 40]. So, we wished to validate the above fact by evaluating the endogenous expression of L1ORF2 and L1ORF1 proteins in somatic and germ line tissues with the generated antibodies. The amino acid stretch from the RT domain used to generate antibody showed significant similarity between human, mice and rat (Fig. 1b). It indicates that the antibody generated using human RT fragment can detect L1ORF2p in mouse and rat. Hence, we wanted to explore the endogenous expression of L1ORF2p in mouse and rat tissues. Tissue lysate from kidney, liver, ovary and testis were prepared, and Western blotting was performed with anti-hL1RT_EH_ antibody for L1ORF2p detection (Fig. 3a and b, upper panel). As expected, the somatic tissues of mice and rat (kidney and liver) did not show any expression of L1ORF2p. In contrast, the germ tissues (ovary and testis) showed robust expression of L1ORF2 protein at around 150 kDa (Fig. 3a and b, upper panel). All the tissues were also assayed for L1ORF1p expression; the mice and rat germ tissues (testis and ovary) showed significant expression of ORF1p (Fig. 3a and b, middle panel), whereas no expression was detected in somatic tissues (kidney and liver) (Fig. 3a and b, middle panel). GAPDH was used as a loading control for both mouse and rat tissues (Fig. 3a and b, lower panel).

Next, we performed immuno histochemistry (IHC) to complement our Western blotting result as well as to detect which group of cells express L1ORF2p protein in mice and rat (Fig. 3c and d). Kidney, liver, testis and ovary tissues were selected both from mouse and rat to see the expression of ORF2p via IHC. The morphology of all the tissue sections was first checked by eosin hematoxylin staining (Fig. 3c and Fig. 3d, upper panel). The IHC analysis showed that both testis and ovary are expressing significant amounts of LIORF2p (Fig. 3c and Fig. 3d, lower panel). All the tissues were also checked for ORF1p expression; the mice and rat germ tissues (testis and ovary) showed significant expression of ORF1p (Fig. 3c and Fig. 3d, middle panel), whereas no or significantly less expression was detected in somatic tissues (kidney and liver) (Fig. 3a and b).

### Detection of L1ORF2p in OSCC

**Fig. 4:**
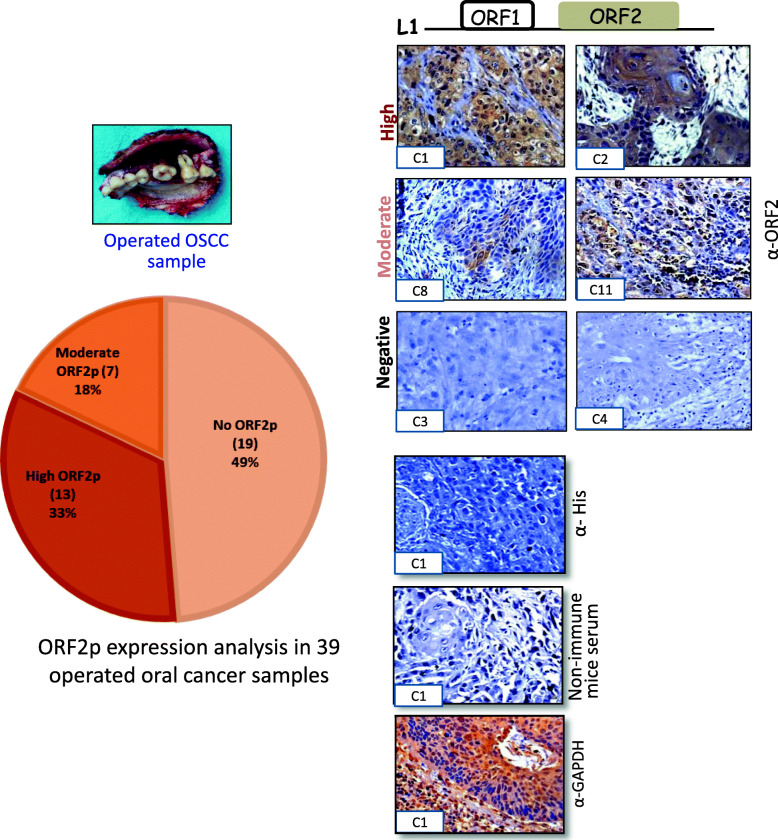
Immuno-peroxidase detection of L1ORF2p expression in post-operative OSCC samples: Immunohistochemistry with anti-L1ORF2p was performed in total 39 post-operatives OSCC. Samples exhibit high (C1 and C2), moderate (C8, C11) and no expression (C3 and C4) (two representatives from each group are shown); IHC staining of the rest of the samples are shown in the supplementary figure. Immuno-staining with anti-His and non-immune mice sera didn’t show any signal. Staining with anti-GAPDH served as a positive control. Images were taken at 40X magnification. The Pie diagram showed more than 50% post-operative OSCC samples expressed a significant amount of L1ORF2p

**Fig. 5:**
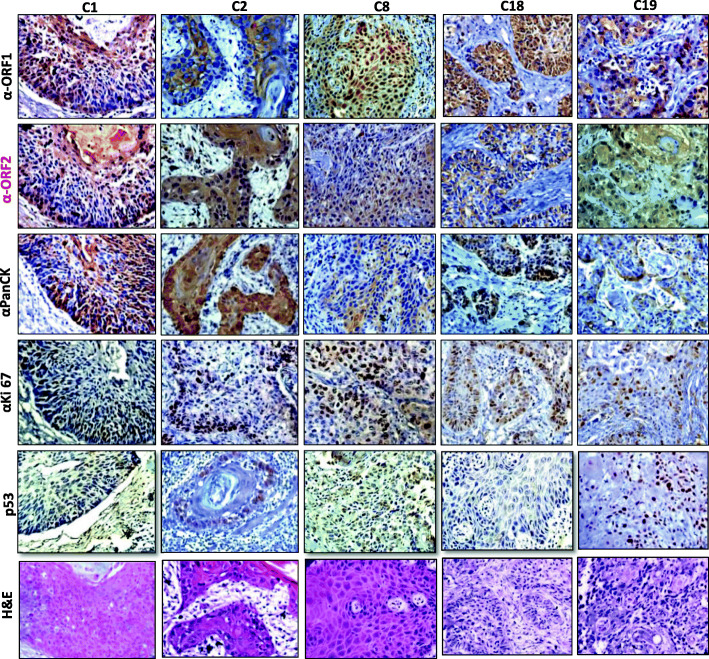
Immuno-peroxidase detection of L1ORF1p, L1ORF2p, Pan-CK, Ki-67 and p53 in operated OSCC samples. IHC staining of five samples (C1, C2, C8, C18, and C19) using all five antibodies are shown. The samples were also stained with hematoxylin and eosin

We next examined the L1ORF2p expression in post-operative OSCC samples. Recently, in a pilot study with a limited number of samples (*n* = 15), we showed significant expression of L1ORF1p in this particular cancer [27]. Here we performed IHC analysis on thirty-nine post-operational oral cancer samples and investigated the expression of L1 ORF1 and ORF2 using our in-house L1ORF1p and L1ORF2p antibodies. The detailed information regarding patients and collected samples are shown in Table 1. First, the neoplastic nature of all cancer samples used in this study was confirmed by hematoxylin and eosin staining (Supplementary Fig. 3). Our IHC analysis with anti-L1hORF2p showed almost 51% (twenty samples) samples were ORF2 positive, and no significant expression was observed in nineteen samples (Fig. 4, Fig. 5 and Supplementary Fig. 4). Careful analysis of the IHC data showed that the expression of ORF2p was very high in thirteen samples (33%), whereas seven samples (18%) showed a moderate level of expression (Fig. 4). The IHC experiments performed with non-immune mouse serum and anti-His primary antibody did not show any signal, thus were treated as negative controls (Fig. 4). The IHC with GAPDH antibody showed significant expression and was treated as a positive control (Fig. 4).

**Table 1:** The details of patients used in this study

Sl.No.	Age	Sex	Site of TUMOR	Grade	P TNM	L1 ORF1p	p53	L1 ORF2p
C1	39	M	Left Upper Alveolus	Moderately Differentiated Squamous Cell Carcinoma	T4A N1	High	Negative	High
C2	56	F	Left Buccal Mucosa	Well Differentiated Squamous Cell Carcinoma	T2 N0	High	Positive	High
C3	27	M	Left Buccal Mucosa	Well Differentiated Squamous Cell Carcinoma	T4A N2B	Negative	Negative	Negative
C4	38	M	Tongue	Moderately Differentiated Squamous Cell Carcinoma	T2 N2C	Negative	Positive	Negative
C5	33	M	Left Buccal Mucosa	Moderately Differentiated Squamous Cell Carcinoma	T4A N2B	Negative	Positive	Negative
C6	60	M	Left Buccal Mucosa	Well Differentiated Squamous Cell Carcinoma	T2 N0	High	Negative	High
C7	45	M	Left Upper Alveolus	Well Differentiated Squamous Cell Carcinoma	T3 N2B	Negative	Negative	Negative
C8	75	F	Left Buccal Mucosa	Well Differentiated Squamous Cell Carcinoma	T4A N0	Moderate	Negative	Moderate
C9	50		Left Buccal Mucosa	Moderately Differentiated Squamous Cell Carcinoma	T4A N2B	Moderate	Positive	Moderate
C10	33	M	Left Buccal Mucosa	Moderately Differentiated Squamous Cell Carcinoma	T4A N0	Negative	Negative	Negative
C11	28	F	Tongue	Poorly Differentiated Squamous Cell Carcinoma	T2N2C	Moderate	Negative	Moderate
C12	40	M	Left Buccal Mucosa	Well Differentiated Squamous Cell Carcinoma	T3 N0	Negative	Negative	Negative
C13	43	M	Left Buccal Mucosa	Moderately Differentiated Squamous Cell Carcinoma	T4A N2B	Negative	Negative	Negative
C14	38	M	Right Lower Alveolus	Well Differentiated Squamous Cell Carcinoma	T4A N0	Negative	Negative	Negative
C15	37	M	Tongue	Moderately Differentiated Squamous Cell Carcinoma	T4aN2Mx	Moderate	Positive	Moderate
C16	42	M	Left Buccal Mucosa	Moderately Differentiated Squamous Cell Carcinoma	T4A N1	Negative	Negative	Negative
C17	28	M	Lower lip	Moderately Differentiated Squamous Cell Carcinoma	T2N2C	Moderate	Negative	Moderate
C18	60	M	Tongue	Moderately Differentiated Squamous Cell Carcinoma	T2 N2B	High	Negative	High
C19	46	M	Left Buccal Mucosa	Moderately Differentiated Squamous Cell Carcinoma	T4A NO	High	Positive	High
C20	38	M	Tongue	Well Differentiated Squamous Cell Carcinoma	T4A N1	High	Positive	High
C21	50	M	Right Buccal Mucosa	Moderately Differentiated Squamous Cell Carcinoma	T2 N0	Negative	Positive	Negative
C22	34	M	Right Buccal Mucosa	Moderately Differentiated Squamous Cell Carcinoma	T3 N0	High	Positive	High
C23	40	M	Right Buccal Mucosa	Moderately Differentiated Squamous Cell Carcinoma	T4A NO	High	Negative	High
C24	40	M	Left Buccal Mucosa	Well Differentiated Squamous Cell Carcinoma	T4A N2B	High	Positive	High
C25	47	M	Right Buccal Mucosa	Moderately Differentiated Squamous Cell Carcinoma	T4A N0	Negative	Negative	Negative
C26	37	M	Right Buccal Mucosa	Moderately Differentiated Squamous Cell Carcinoma	T3N2B	Negative	Positive	Negative
C27	59	F	Left Buccal Mucosa	Moderately Differentiated Squamous Cell Carcinoma	T4A N0	Negative	Negative	Negative
C28	40	M	Left Buccal Mucosa	Moderately Differentiated Squamous Cell Carcinoma	T4A N2B	Moderate	Negative	Moderate
C29	51	M	Right Buccal Mucosa	Moderately Differentiated Squamous Cell Carcinoma	T2N0	Negative	Negative	Negative
C30	50	M	Left Buccal Mucosa	Poorly Differentiated Squamous Cell Carcinoma	T4A N2B	Negative	Negative	Negative
C31	50	M	Lower Lip	Moderately Differentiated Squamous Cell Carcinoma	T4A N2C	Negative	Negative	Negative
C32	44	M	Left Buccal Mucosa	Moderately Differentiated Squamous Cell Carcinoma	T4AN0	High	Positive	High
C33	52	M	Tongue	Well Differentiated Squamous Cell Carcinoma	T4AN2B	High	Positive	High
C34	40	M	Left Buccal Mucosa	Moderately Differentiated Squamous Cell Carcinoma	T2N0	Negative	Negative	Negative
C35	34	M	Right Buccal Mucosa	Moderately Differentiated Squamous Cell Carcinoma	T4AN0	High	Positive	High
C36	43	M	Right Buccal Mucosa	Moderately Differentiated Squamous Cell Carcinoma	T1N0Mx	Negative	Negative	Negative
C37	44	M	Tongue	Moderately Differentiated Squamous Cell Carcinoma	T3N2M_X_	High	Negative	High
C38	43	M	Right Buccal Mucosa	Moderately Differentiated Squamous Cell Carcinoma	T4AN1	Moderate	Negative	Moderate
C39	46	M	Left Buccal Mucosa	Moderately Differentiated Squamous Cell Carcinoma	T3N2B	Negative	Positive	Negative

We next investigated all of the samples for expression of hL1ORF1p. The IHC experiment showed that all the twenty samples which were positive for the hL1ORF2p also showed a significant amount of hL1ORF1p expression (Fig. 5 and Supplementary Fig. 5).

Previous studies reported increased L1ORF1p expression is more common in p53 deficient human cancers [25, 41, 42]. By employing IHC, we have analyzed aberrant p53 expression in all the thirty-nine post-operative cancer samples (Fig. 5 and Supplementary Fig. 6). A significant high expression of p53 was observed in nearly 40% samples (15 out of 39 samples), of which ten samples showed significantly high expression of L1 ORF2p. Careful observation showed p53 present only in the nucleus, whereas L1-ORF2p present mainly in the cytoplasm and they overlapped with each other (Supplementary Fig. 7). Normal adjacent tissues didn’t show L1ORF2p and p53 expression (Supplementary Fig. 7). In parallel, the IHC analysis was also performed with anti-PanCK (a marker for epithelial cell) and anti-Ki67 (markers for proliferative cells) for all 39 cancer samples (Fig. 5). The expression of proteins in all the samples indicated that the post-operational tissues were enriched for cancer cells of epithelial origin.

**Fig. 6:**
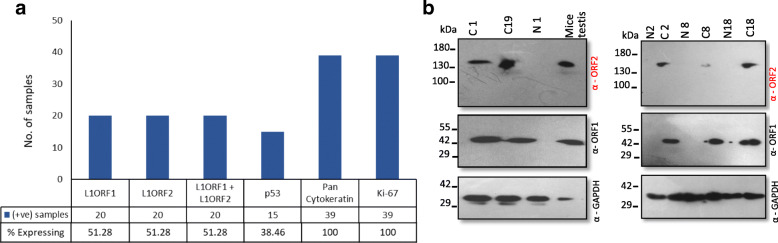
Expression analysis of L1ORF1p and L1ORF2p in OSCC(**a**) Bar diagram showing the percent expressing L1ORF1p, L1ORF2p, L1ORF1p + L1ORF2p, p53, Pan-CK and Ki67. **b** Immunoblot analysis of L1ORF1p and L1ORF2p expression in paired normal cancer samples (C1, C2, C8, C18, and C19). Anti-L1ORF2p detects a distinct band at around 150 kDa corresponds to the MW of L1ORF2p in all the cancer tissues but not in paired normal. The same blot was re-probed with anti-L1ORF1p and anti-GAPDH (original blots are attached in supplementary file)

To complement the IHC results, we performed an immunoblotting experiment to determine the expression of L1ORF1p and L1ORF2p. Three paired samples (normal-cancer pairs) and two samples with only cancer tissues which showed L1-ORFs expression by IHC were tested (Fig. 6). All five cancer tissues showed a distinct ORF2p band corresponding to150 kDa, the MW of human L1ORF2p. Among the five samples, sample C19 showed the highest expression, while sample C1 showed the lowest expression of L1ORF2p. Lysate obtained from adjacent normal tissues did not show any expression of L1ORF2p. The testis lysate from mice that showed L1ORF2p at around 150 kDa was used as a positive control (Fig. 6b). Immunoblot analysis with anti-hL1ORF1p showed very high expression of L1ORF1p in all five cancer tissues, and no expression was detected in adjacent normal tissues. The GAPDH immunoblotting was used to check the quality of the lysate.

## Discussion

Several studies have reported increased L1 activity in the germ cells and in cancers [22–24, 29, 40]. In all these studies, our understanding of L1ORF2p expression is limited. Although L1ORF1p is readily detectable [25, 27, 32], L1ORF2p is very challenging to detect in vitro and in vivo. As the recombinant L1ORF2p doesn’t express well, short synthetic peptides from the entire length of L1ORF2p protein sequence have been mainly used for the generation of L1ORF2p antibody [28, 29, 33–35]. In the past, a number of polyclonal and monoclonal antibodies against human and mouse L1-ORF2p have been reported [28, 29, 32–35]. However, questions have been raised about the specificity of the reported ORF2p antibodies. Here we found an eighty amino acids stretch at the RT domain [fragment name: hRT_EH_, ORF2p amino acid position 479–558, nucleotide position in L1 RP (Accession No.AF148856) [37] flanked by natural restriction enzymes *EcoRI* and *HindIII* that when cloned and expressed in a bacterial expression vector showed significantly high expression. In silico folding of the L1ORF2 protein revealed this particular 80 amino acid stretch protruding outside, making it a suitable epitope for antibody generation. Although the recombinant hL1ORF2RT_EH _fragment peptide formed inclusion bodies, we were successful in purifying and folding it to its native conformation. The purified hL1RTORF2_EH_ fragment induced an adequate antibody response when injected into mice. The whole serum and purified IgG fraction from immunized mice showed a discrete single band at around 150 kDa, which corresponds to the MW of hL1ORF2p, suggesting that the antibody is highly specific for detection of L1ORF2 protein.

The eighty amino acids stretch used to make ORF2p antibody showed strong sequence conservation at that particular stretch when aligned with mice and rat L1ORF2 sequences. Along with this, we found strong immuno reactivity of this antibody with the mice and rat endogenous L1ORF2p expressed in the germ tissues. Earlier Branciforteet et al. [40] reported two mouse-specific ORF2p antibody; both generated using carboxy-terminal two-third of mouse L1ORF2 protein as antigen. Although the generated antibody showed strong immuno reactivity against the recombinant antigen produced in *E. coli*, no immuno reactivity was detected against endogenous L1ORF2p expressed in mouse testis and ovary. This suggests that the epitope used to generate antibodies against mouse L1ORF2p might be buried inside in the native full-length L1ORF2p, and hence no immuno reactivity was detected [40].

Recently, we have reported a human L1ORF1p antibody which was generated using the RRM domain of human L1ORF1p [26]. Careful observation revealed that amino acid sequences in the RRM domain of the human L1ORF1p are much conserved when compared with mouse and rat L1ORF1 RRM domain amino acids [43, 44]. Here we showed that human L1 ORF1p antibody generated against the RRM domain of human L1ORF1p elicitated a strong immune response against mouse and rat L1ORF1p expressed in the germ tissues. Thus, both the in-house antibodies (anti-hL1ORF1p and anti-hL1ORF2p) detect L1 proteins in all three species (human, mouse and rat) suggesting that these could be useful reagents to study the biology of L1 retrotransposons.

Several studies have demonstrated elevated L1 retrotransposon activity in cancers [23–27, 45–48]. L1 promoters, which are heavily methylated in normal tissue to restrict L1 retrotransposition, are often hypomethylated in tumours leading to L1 retrotransposon activity [49–54]. Whole-genome and targeted sequencing approaches have shown a significant L1 retrotransposon copy number increase in cancer tissues compared to the normal counterpart [23, 24, 42, 46]. Further characterization revealed that the increased copy number of L1 retrotransposons in cancer is the result of active retrotransposition by the TPRT mechanism where both the L1 encoded proteins (L1ORF1p and L1ORF2p) are strictly required [24, 48]. In a pioneer study, Rodic et al. demonstrated L1ORF1p expression is common, and nearly half of the cancers expressed L1ORF1p as evident by IHC analysis [25]. Attempts to show the L1ORF2 protein expression in cancer tissues are minimal due to the non-availability of an effective L1ORF2p antibody. Here, we have successfully generated a specific L1ORF2p antibody. By employing IHC and immunoblotting, we analysed the expression of L1ORF2p in 39 post-operative oral cancer samples, of which more than 50 % samples showed significant L1ORF2p expression. In parallel, we also found L1ORF1p expression in all the samples that showed L1ORF2p expression. Previous studies reported that an increased presence of L1ORF2p in the nucleus is associated with advanced stages of cancer [32, 34]. The studies also reported that in some cancers, the expression of L1ORF2p is very high at the early transformation stage [32]. Our data showed among the L1ORF2p positive samples (20 out of 39), some are expressing a very high amount of L1ORF2p. When we compared the amount of ORF2p expression with the grades and TNM staging, no correlation was observed.

The wild type p53 has very short half-life generally localizes in the cell nucleus in very small quantities that it cannot be detected by routine IHC [55, 56]. Missense mutations often increase the half-life and the quantity of p53 expression, allowing its detection by IHC [57]. However, some tumors are frequently immuno-positive for p53 in the absence of mutation [58]. Potential mechanisms for accumulation of non-mutant p53 include stabilization of p53 protein by binding to viral or cellular protein and DNA damage by the chemical and physical genotoxic agent [57]. Thus IHC is used as a screening test before DNA sequencing to find out missense mutation or over expression of wild type p53 gene for few cancers. Previous studies showed that in cancer, the increased L1ORF1p expression often correlates with p53 mutations and aberrant p53 expression [25, 27, 41]. Wylie et al. [41] demonstrated that the loss of p53 is strongly associated with elevated retrotransposon activity. In the 39 OSCC samples, p53 positive staining was found in nearly 40% of the tumors by IHC. Further study is required to understand whether mutations in the p53 gene have any role in elevated L1 protein expression in OSCC cancer.

In summary, we have successfully developed a polyclonal L1ORF2p antibody, which is very specific and detects L1ORF2p in post-operative oral cancer tissue. As the epitope used to generate L1ORF2p antibody is highly conserved, we found the antibody is equally useful to detect the same protein in mouse and rat germ tissues. The novel L1ORF2p antibody reported in this study will serve as a useful tool for oral cancer studies and diagnostic applications. Further study is required to understand why L1 activity is deregulated in OSCC and how it contributes to the progression of this particular cancer.

## Conclusions

The L1 retrotransposons show very high activity in germ tissues and many cancers. It expresses two proteins (L1ORF1p and L1ORF2p), both are required for the retrotransposition of L1 mRNA. Previous studies demonstrated that almost half of the cancers show the expression of L1ORF1p. However, due to the unavailability of an effective L1ORF2p antibody, the detection of L1ORF2p has not been reported. In this study, we have developed a very specific polyclonal L1ORF2p antibody and showed its robust expression in post-operative oral cancer samples. As the selected epitope used to generate L1ORF2p antibody is highly conserved, we found the antibody is equally useful to detect the same protein in mouse and rat germ tissues. The novel L1ORF2p antibody reported in this study will serve as a useful tool for oral cancer studies and diagnostic applications. Further study is required to understand why L1 activity is deregulated in OSCC and how it contributes to the progression of this particular cancer.

## Supplementary Information

**Additional file 1.**

## Data Availability

The data set generated and/or analyzed during the current study is available in the manuscript’s result and supplementary sections. No data in this study has been deposited in any web link.

## Abbreviations

%: Percentage
μg: microgram
AIIMS: All India Institute of Medical Science
bp: base pair
BSA: Bovine Serum Albumin
CC: Coiled Coil
CTD: Carboxy Terminal Domain
DAB: 3-3´- Diaminobenzidine tetrahydrochloride (DAB substrate)
DMEM: Dulbecco’s Modified Eagle Medium
DNA: Deoxyribonucleic Acid
DPX: Dibutylphthalate Polystyrene Xylene
ECL: Enhanced chemiluminescence
EDTA: Ethylene Diamine Tetra-Acetic Acid
EN: Endonuclease
FBS: Fetal Bovine Serum
FFPE: Formalin-Fixed Paraffin Embedded
GAPDH: Glyceraldehyde 3-phosphate dehydrogenase
EGFP: Enhanced Green Fluorescent protein
H&E staining: Haematoxylene and Eosin
HRP: Horseradish peroxidase
hrs: hours
IHC: Immunohistochemistry
IHEC: Institutional Human Ethics Committee
IPTG: Isopropyl β-D-1-thiogalactopyranoside
kb: kilo bases
kDa: kilo Dalton
L1-RNPs: LINE-1 ribonucleoproteins
LB: Luria-Bertani
LINE-1: Long INterpersed Element-1
ml: millilitre
mM: mill molar
mRNA: Messenger RNA
MW: Molecular Weight
NaCl: Sodium Chloride
NBF: Neutral Buffered Formalin
ng: Nanogram
NIMHANS: National Institute of Mental Health and Neurosciences
O/N: overnight
^o^C: degree centigrade
ORF: Open Reading Frame
OSCC: Oral Squamous Cell Carcinoma
PBS: Phosphate Buffered Saline
PBS-T: Phosphate Buffered Saline-Tween
RNP: Ribonucleoprotein
RRM: RNA Recognition Motif
RT: Reverse transcriptase
SDS-PAGE: Sodium Dodecyl Sulphate-Polyacrylamide Gel Electrophoresis
SINE: Short INterspersed Elements
SVA: SINE-R/VNTR/Alu
TBST: Tris Buffered Saline-Tween
TP53: Tumor Protein53
TPRT: Target-Primed Reverse Transcription
TSD: Target Site Duplication
UTR: Untranslated region
WGS: Whole Genome Sequencing
