# Supplementary material for: Robust expression of LINE-1 retrotransposon encoded proteins in oral squamous cell carcinoma
Source: BMC Cancer. 2021 May 27;21:628. doi: 10.1186/s12885-021-08174-z (PMC8161598; doi:10.1186/s12885-021-08174-z)
Supplement: Supplementary file 1 — Additional file 1. [file 12885_2021_8174_MOESM1_ESM.pdf]

Supplementary Figures and text

Title: Robust expression of LINE-1 retrotransposon encoded proteins in oral squamous cell carcinoma

Koel Mukherjee[1], Debpali Sur[1±], Abhijeet Singh[2±], Sandhya Rai[1], Neeladrisingha Das[1], Rakshanya Sekar[4],Srinu Narindi[3],Vandana Kumar Dhingra[2], Bhinyaram Jat[2], K V Vinu Balraam[3], Satya Prakash Agarwal[2], Prabhat Kumar Mandal[1*]

1. Department of Biotechnology, IIT Roorkee, Roorkee, Uttarakhand, India.
2. Department of Head-Neck Surgery and Oncology, AIIMS Rishikesh, Rishikesh Uttarakhand, India.

3.Military Hospital, Roorkee, Uttarakhand, India.

4. School of Biosciences and Technology, Vellore Institute of Technology, Vellore, Tamil Nadu, India.

kmukherjee@bt.iitr.ac.in
debpali.rony@gmail.com
abhijeetsingh89@gmail.com
srai@bt.iitr.ac.in
ndas@bt.iitr.ac.in
rakshanya.Sekar@anu.edu.au
srinunarindi2632@gmail.com
vandana.numed@aiimsrishikesh.edu.in
bhinyaram.jat@gmail.com
vbalraam@gmail.com
drspagarwal06@gmail.com
prabhat.mandal@bt.iitr.ac.in

± These authors contributed equally

*Correspondence should be addressed to mandal.prabhat@gmail.com or prabhat.mandal@bt.iitr.ac.in.

Supplementary Figures :

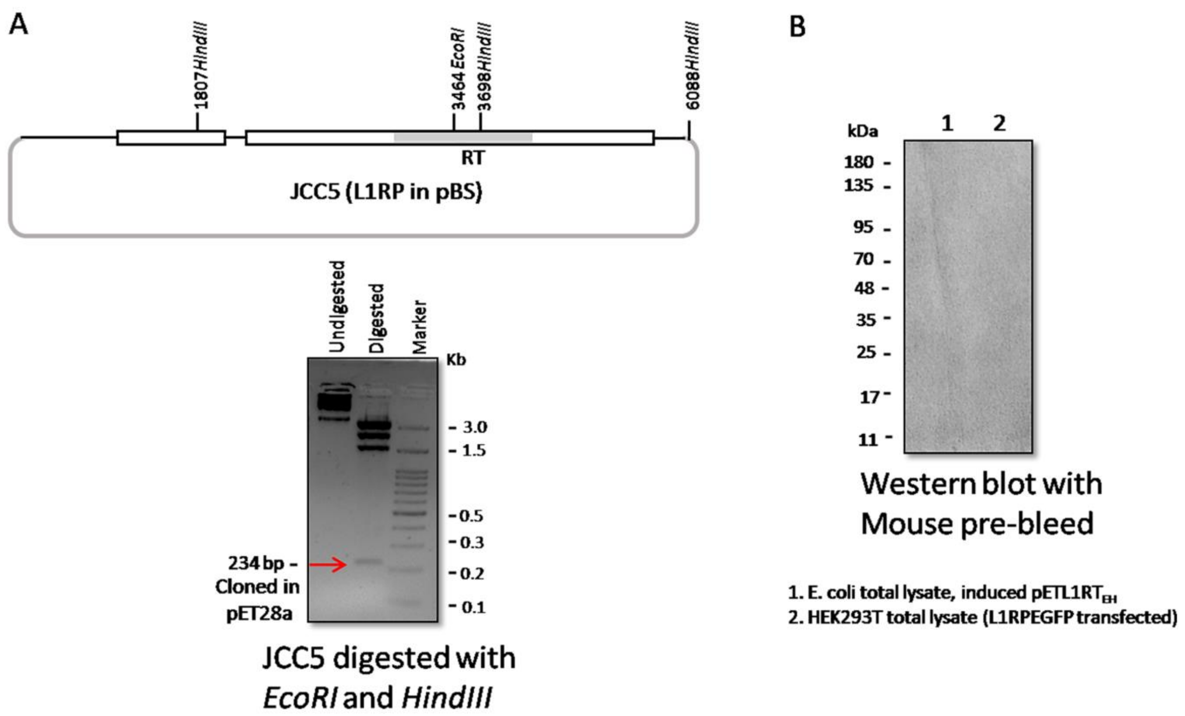

Supplementary figure 1: (A) Schematic map of JCC5 (L1RP in pBSKS plasmid) [37] and its restriction digestion with *EcoRI* and *HindIII* restriction enzymes. The digested product was resolved in 1.5% agarose gel. The 234 bp RT fragment (marked by arrow) was subcloned in Pet28a bacterial expression vector. (B) Western blot of L1RT$_{EH}$ (immunogen) and full length exogenous L1ORF2p with non-immune mice sera.

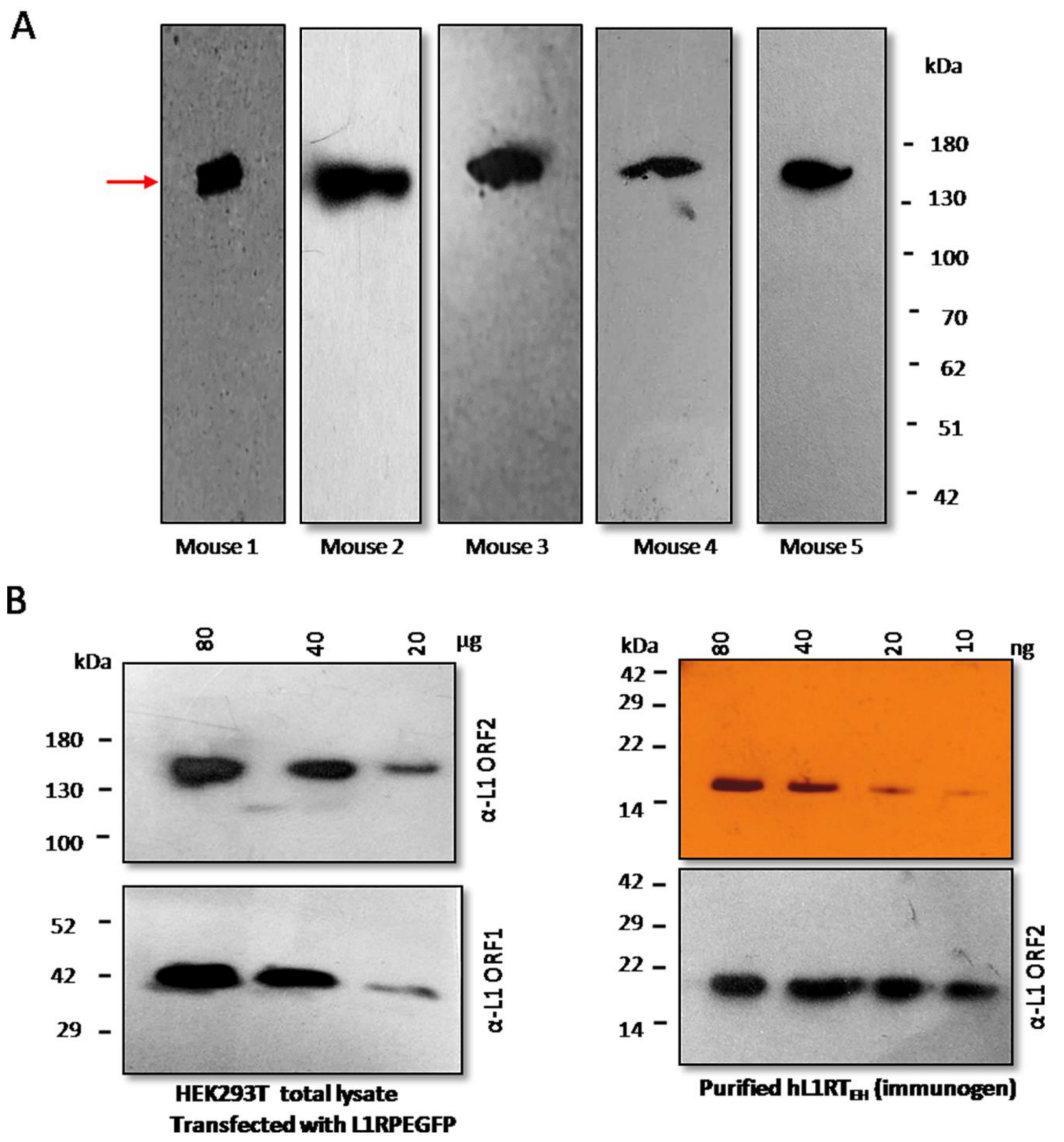

Supplementary figure 2: (A) Immune sera from five different mice were checked by Western blot showing a distinct 150 kDa band of exogenous L1ORF2p in HEK293T total lysate transfected with L1RPEGFP [38]. (B) Sensitivity analysis of L1 ORF2p antibody by Western blot and silver staining.

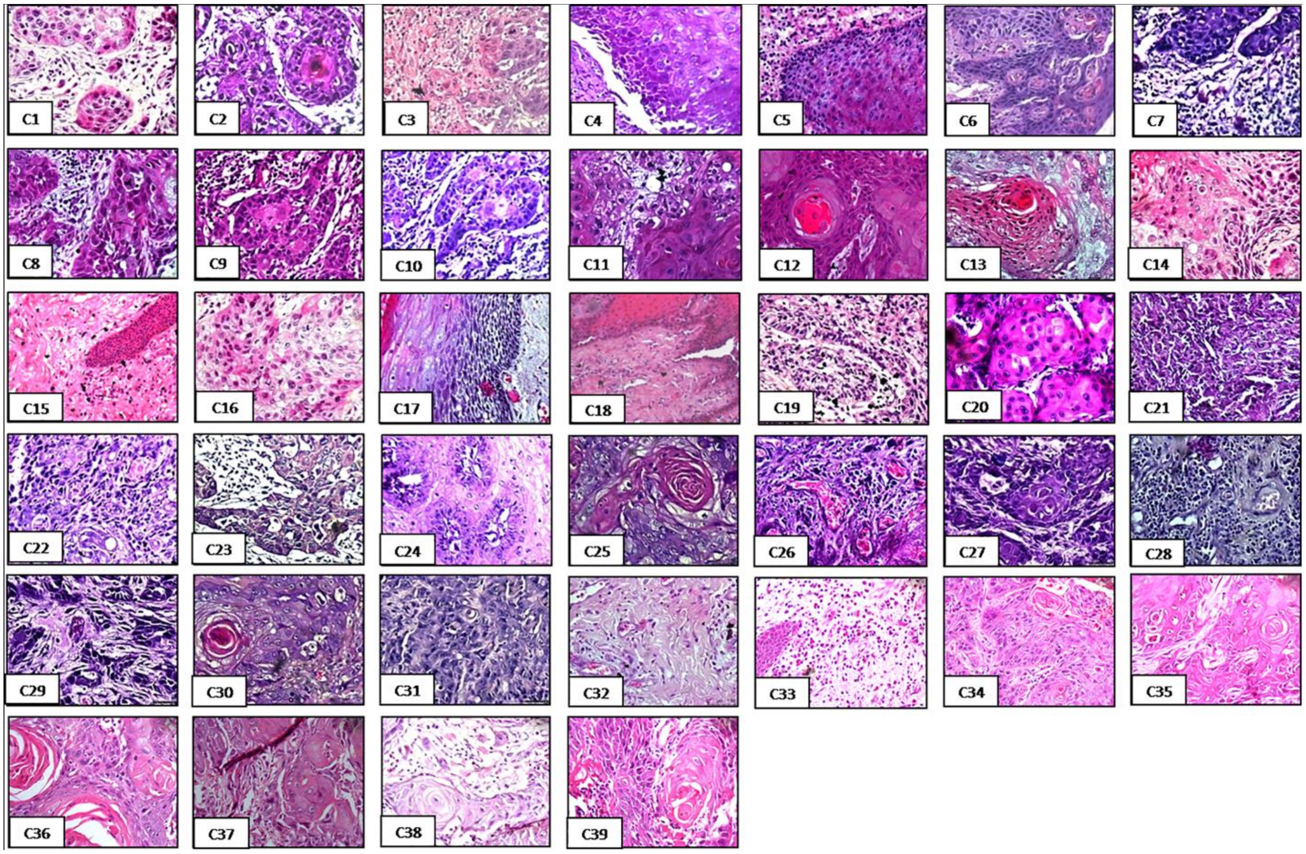

Supplementary figure 3: Hematoxylin-eosin stained section of post-operative OSCC samples.

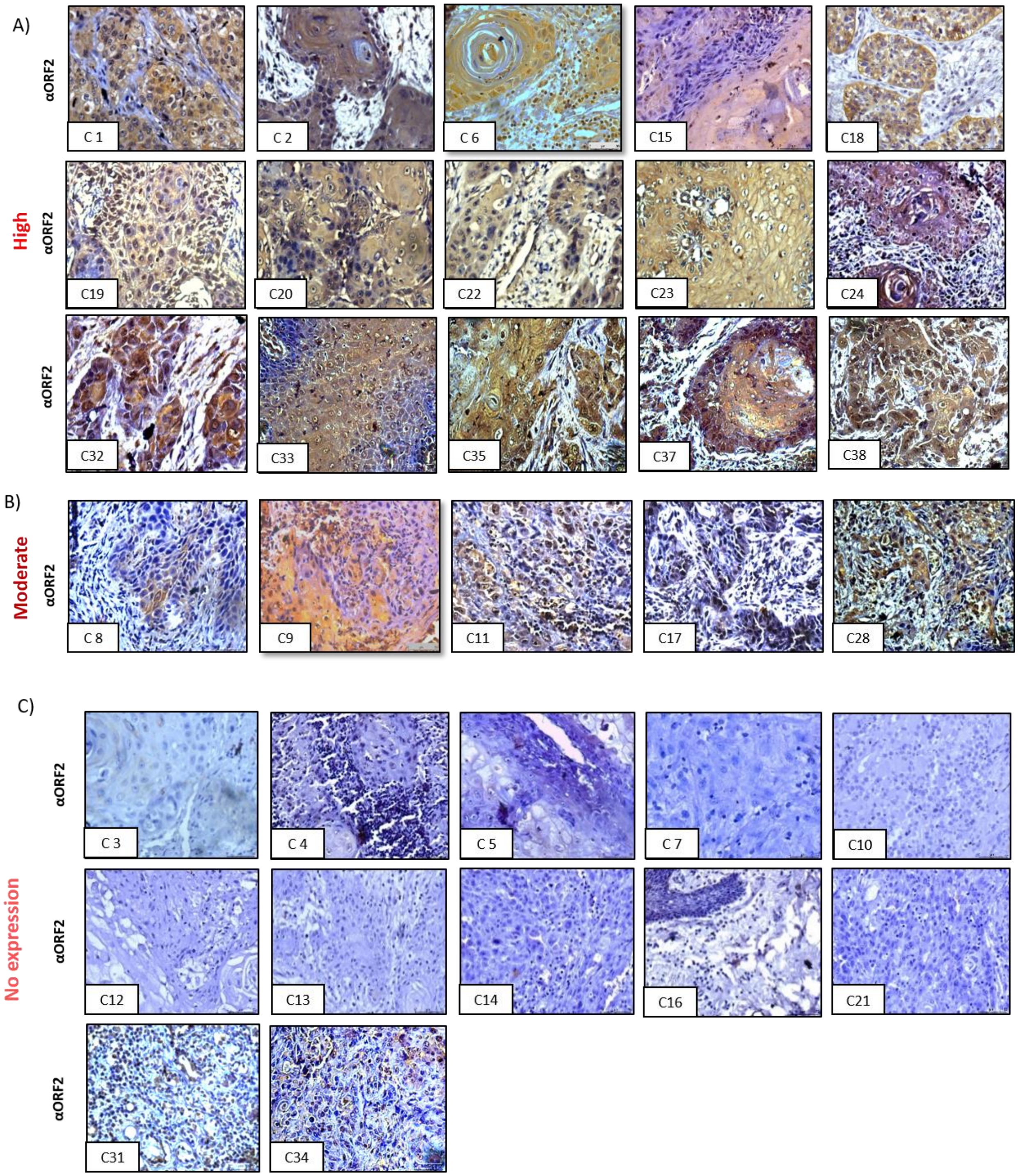

Supplementary figure 4: IHC analysis of L1ORF2p expression in post-operative OSCC samples. (A) High L1ORF2p expression. (B) Moderate expression of L1ORF2p. (C) Less or no expression of L1ORF2p.

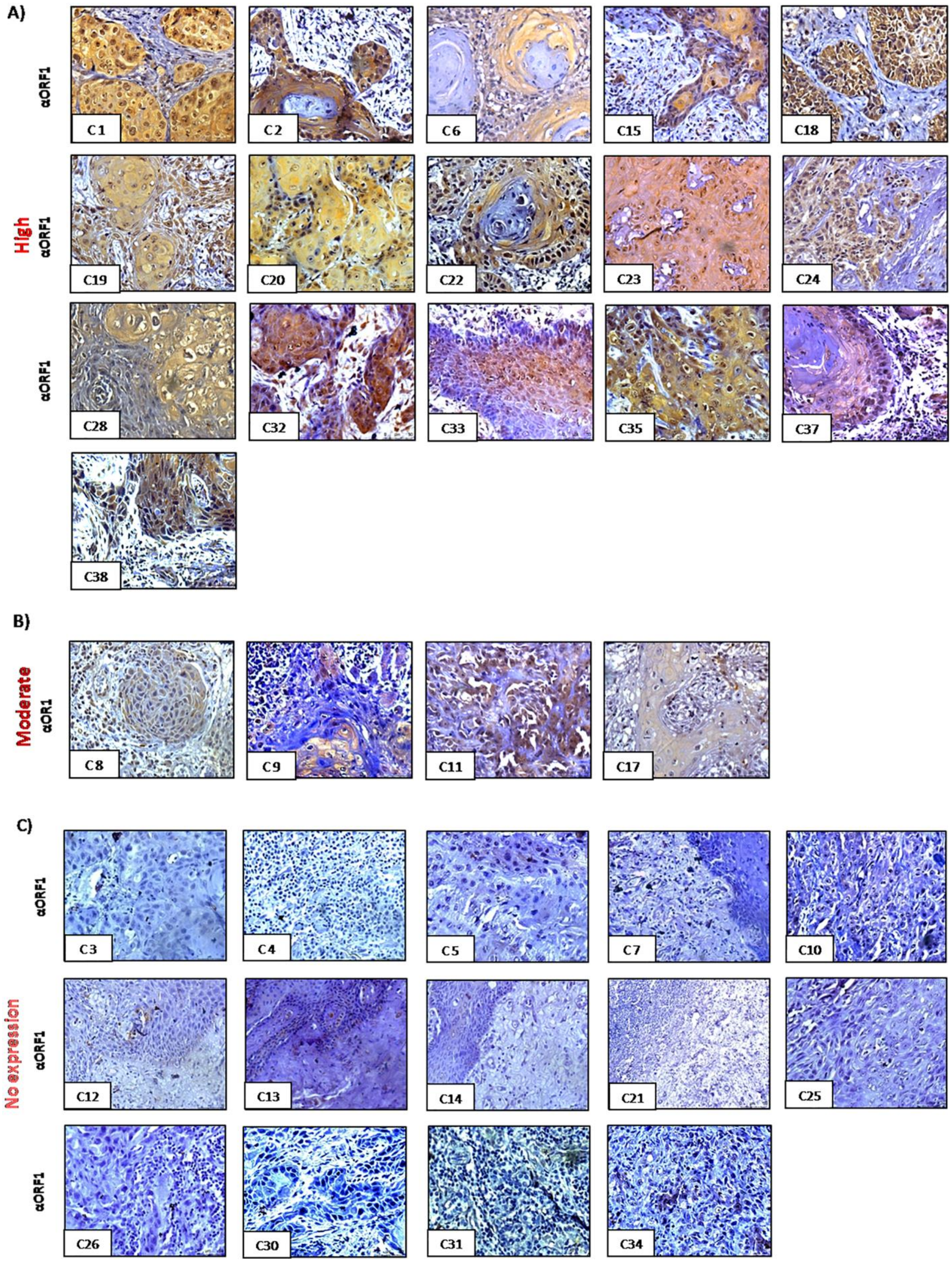
A)
High
αORF1
C1
C2
C6
C15
C18
C19
C20
C22
C23
C24
C28
C32
C33
C35
C37
C38
B)
Moderate
αOR1
C8
C9
C11
C17
C)
No expression
αORF1
C3
C4
C5
C7
C10
C12
C13
C14
C21
C25
C26
C30
C31
C34

Supplementary figure 5: IHC analysis of L1ORF1p expression in post-operative OSCC samples. (A) High L1ORF1p expression. (B) Moderate expression of L1ORF1p. (C) Less or no expression of L1ORF1p.

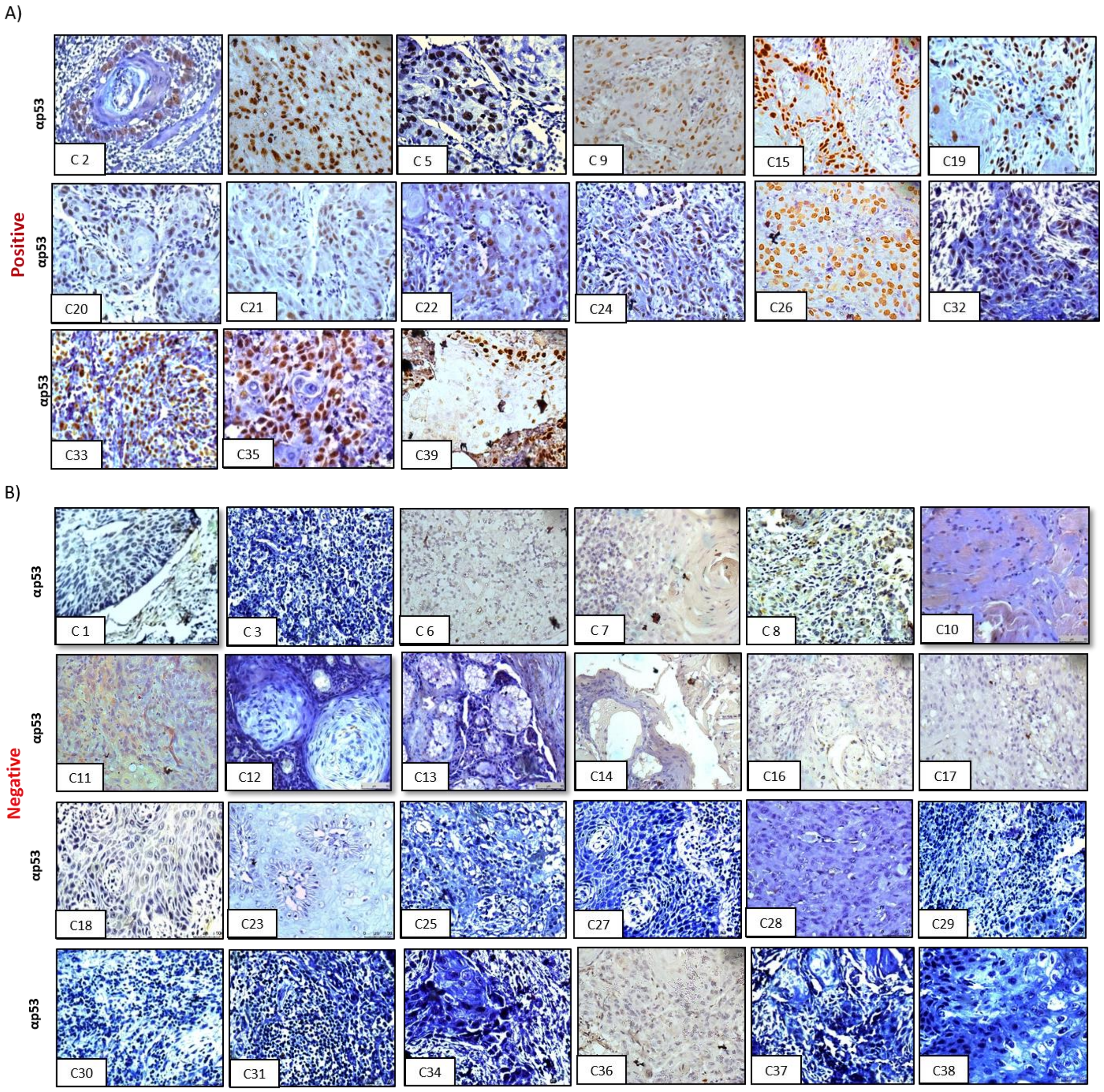

Supplementary figure 6: IHC analysis of p53 expression in post-operative OSCC samples. (A) Samples showing positive p53 expression. (B) Samples showing negative p53 expression.

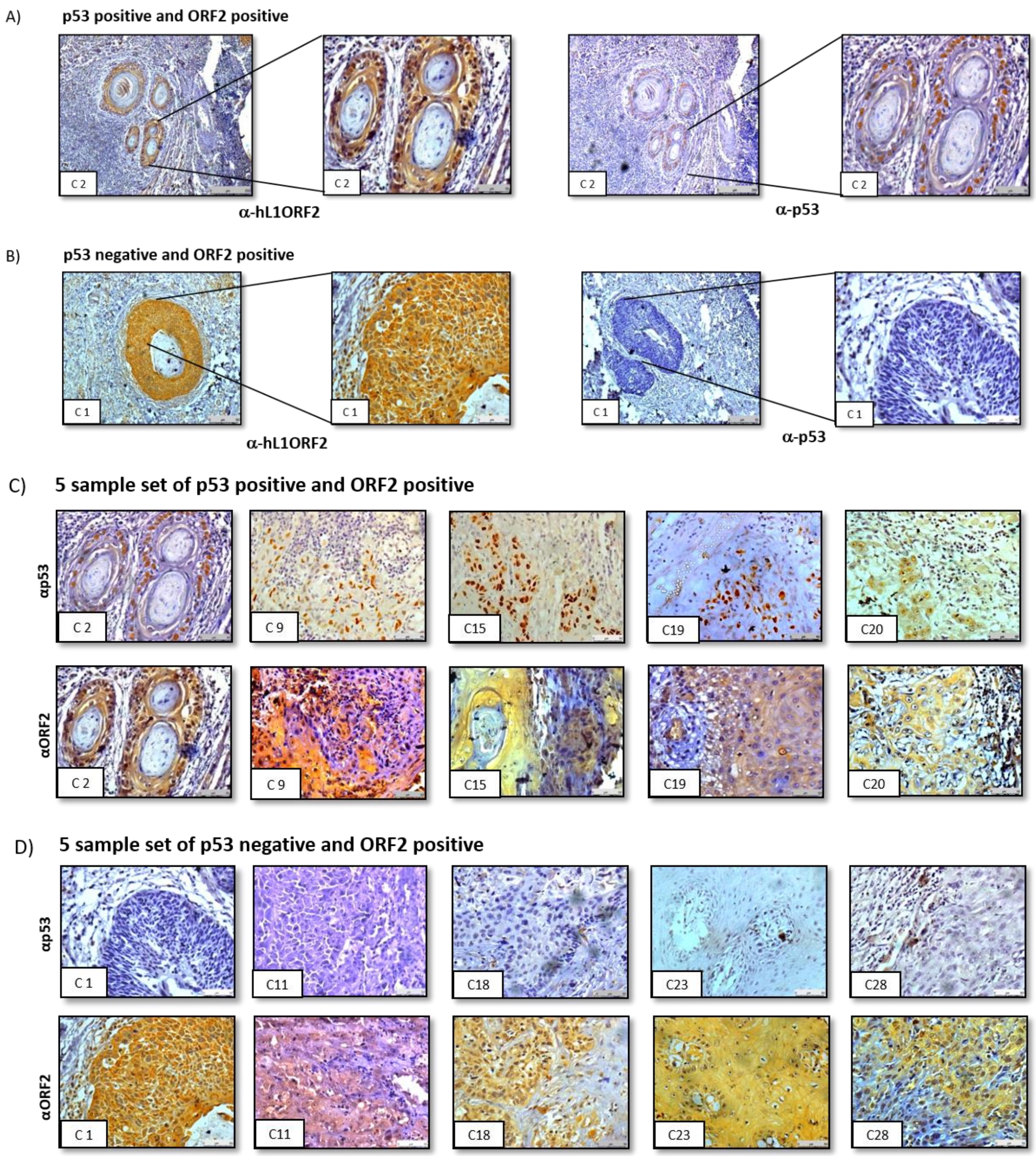

Supplementary figure 7: IHC analysis of L1-ORF2p expression in p53 positive and p53 negative samples of OSCC. (A) IHC analysis of L1-ORF2p expression in p53 positive sample (C2). (B) IHC analysis of L1-ORF2p expression in p53 negative sample (C1). (C) Representative of five samples (C2, C9, C15, C19 and C20) showing L1-ORF2p expression in p53 positive

samples. (D) Representative of five samples (C1, C11, C18, C23 and C28) showing L1-ORF2p expression in p53 negative samples.

Supplementary text:

Cloned in pET 28a between ECOR1 and Hind III:

ATGGGCAGCAGCCATCATCATCATCATCACAGCAGCGGCCTGGTGCCGCGCGGCAG
CCATATGGCTAGCATGACTGGTGGACAGCAAATGGGTCGCGGATCCGAATTCTACC
AGAGGTACAAGGAGGAACTGGTACCATTCCTTCTGAAACTATTCCAATCAATAGAA
AAAGAGGGAATCCTCCCTAACTCATTTTATGAGGCCAGCATCATTCTGATACCAAAG
CCGGGCAGAGACACAACCAAAAAAGAGAATTTTAGACCAATATCCTTGATGAACAT
TGATGCAAAAATCCTCAATAAAATACTGGCAAACCGAATCCAGCAGCACATCAAAA
AGCTTGCGGCCGCACTGGAGCACCACCACCACCACCACTGA

Chromatogram from sanger sequencing of recombinant ORF2 protein:

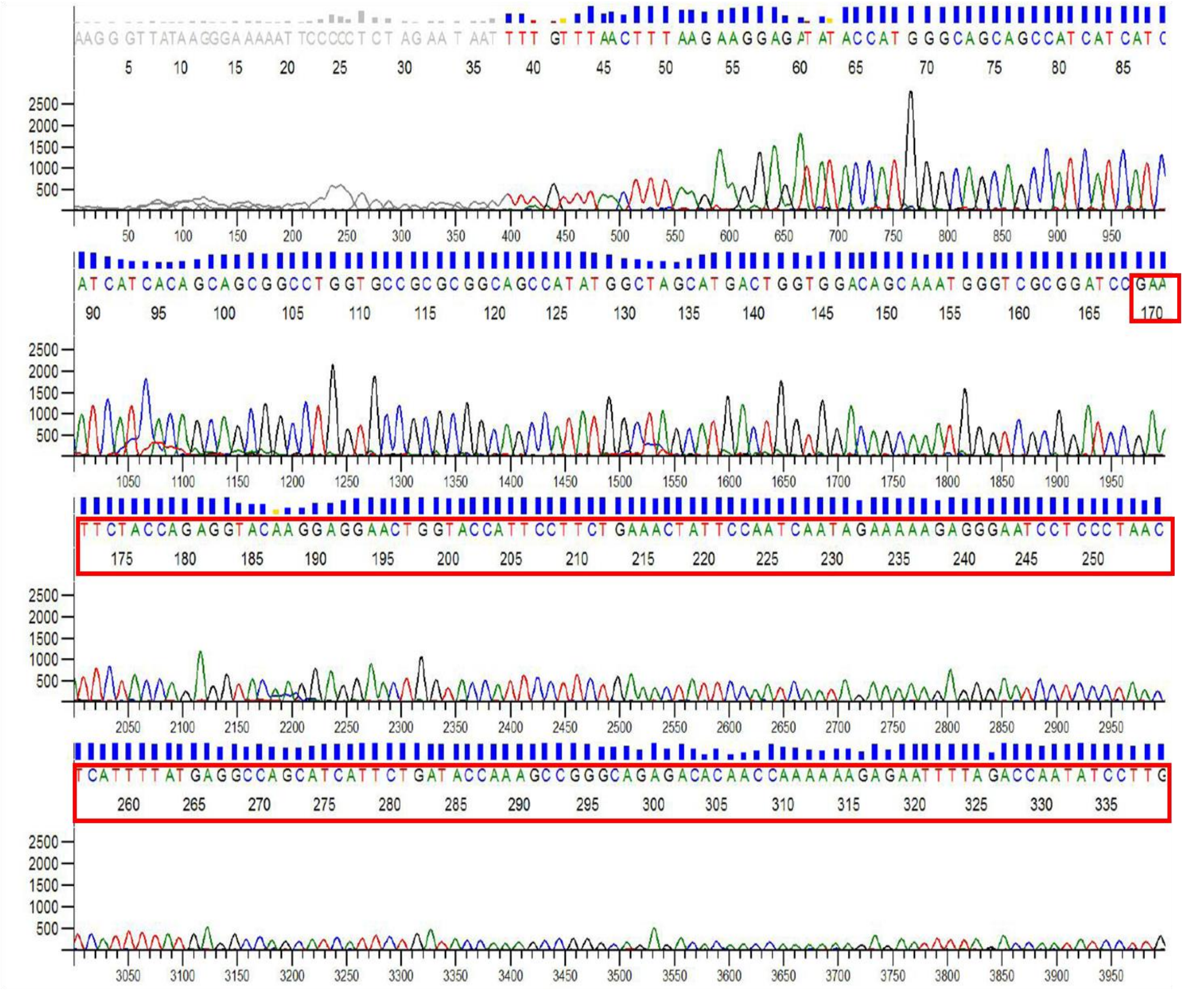

Red box indicates the cloned sequence

Human L1RP (Acc No. AF148856)
Mice L1 (Acc No. M29324.1) (J. Mol. Biol. 196;757-767 ;1987)
Rat L1 (Acc No. DQ100473.1)(Kirilyuk et al. NAR 2008)

>Human L1ORF1 (L1Rp)
MGKKQNRKTGNSKTQSASPPPKERSSSPATEQSWMENDFDELREEGFRRSNYSELREDIQTKGKEVENFEKNLEECITR
ITNTEKCLKELMELKTKARELREECRSLRSRCDQLEERVSAMEDEMNEMKREGKFREKRIKRNEQSLQEIWDYVKRPNL
RLIGVPESDVENGTKLENTLQDIIQENFPNLARQANVQIQEIQRTPQRYSSRRATPRHIIVRFTKVEMKEKMLRAAREKG
RVTLKGKPIRLTADLSAETLQARREWGPIFNILKEKNFQPRISYPAKLSFISEGEIKYFIDKQMLRDFVTTRPALKELLKEALN
MERNNRYQPLQNHAKM
>Mouse L1 ORF1 (GF21)
MAKGKRKNPTNRSQDHSPSSEPRTPTSPNPGHPNTPEKVDVDLKAYLMMMVEDIKKEFNNSLKEIQENTAKELQVLKE
KQENTAKELQVLKEKQENTTKQVEVLIEKQENTSKQVMEMNKTILDLKREVDTIKKTQSEATLEIETLGKKSGTIDASISN
RIQEMEERISGAEDSIENIGTTIKENGKCKKILTQNIQEIQDTMRRPNLRIIGVDENEDFQLKGPANIFNKIIEENFPNLKKE
MPMNIQEAYRTPNRLDQKRNSSRHIIIRTPNALNKDRILKAVREKGQVTYKGKPIRITPDFSPETMKARRAWTDVIQTLR
EHKCQPRLLYPAKLSITIDGETKVFHDKTKFTHYLSTNPALQRIITEKKQYKDGNHALEKTRR
>Rat L1 ORF1
MARGKRRNPSNRNQDCMPSSEPNSPAKTNMEYPNTPEKQDLVSKSYLIMMLEDFKKDMNTLRETQEIINKQVEAYRE
EWQKSLKEFQENTIKQMKELKMEIEAIKKEHMETTLDIENQKKRQGAVDTSFTNRIQEMEERISGAEDSIEIIDSTVKDN
VKRKKLLVQNIQEIQDSMRRSNLRIIGIEESEDSQLKGPVNIFNKIIEENFPNLKKEIPIGIQEAYRTPNRLDQKRNTSRHIIV
KTPNAQNKERILKAVREKGQVTYKGRPIRITPDFSPETMKARRSWTDVIQTLREHKCQPRLLYPAKLSINIDGETKIFHDK
TKFTQYLSTNPALQRIINGKAQHKEASYTLEEARN

CLUSTAL multiple sequence alignment of Human, Mice and Rat L1ORF1 protein sequences.
Yellow shaded part from human L1ORF1 was used to make L1ORF1 specific antibody

```
Human           --MGKKQNRKTGNSKTQSASPPP---KERSSSPATE--QSWMENDFDELREEGFRRSNYS     53
Mouse           MAKGKRKNPTNRSQDHSPSSEPRTPTSPNPGHPNTPEKVDVDLKAYLMMMVEDIKKEFNN     60
Rat             MARGKRRNPSNRNQDCMPSSEPNSPAKTNMEYPNTPEKQDLVSKSYLIMMLEDFKKDMNT     60
                   **::* .. ...   :* *    . .   * *    .   : :  :  *.:::.  .

Human           ELREDIQTKGKEVENFE---------------------KNLEECITRITNTEKCLKELME     92
Mouse           SLKEIQENTAKELQVLKEKQENTAKELQVLKEKQENTTKQVEVLIE---KQENTSKQVME     117
Rat             L-RETQEIINKQVEAYREEWQK------SLKE-----------------FQENTI-----     91
                  :*  :   *:::  .                                  *:

Human           LKTKARE-------------------------LREECRSLRSRCDQLEERVSAMEDEMNE     127
Mouse           MNKTILDLKREVDTIKKTQSEATLEIETLGKKSGTIDASISNRIQEMEERISGAEDSIEN     177
Rat             --KQMKELKMEIEAIKKEHMETTLDIENQKKRQGAVDTSFTNRIQEMEERISGAEDSIEI     149
                  .   :                               *: .* :::***:*. **.::

Human           MKREGKFREKRIKRNEQSLQEIWDYVKRPNLRLIGVPESDVENGTKLENTLQDIIQENFP     187
Mouse           IGTTIKENGKCKKILTQNIQEIQDTMRRPNLRIIGVDENEDFQLKGPANIFNKIIEENFP     237
Rat             IDSTVKDNVKRKKLLVQNIQEIQDSMRRSNLRIIGIEESEDSQLKGPVNIFNKIIEENFP     209
                :    * . *  *   *.:*** * ::* ***:**: *.:  : .   * ::.**:****

Human           NLARQANVQIQEIQRTPQRYSSRRATPRHIIVRFTKVEMKEKMLRAAREKGRVTLKGKPI     247
Mouse           NLKKEMPMNIQEAYRTPNRLDQKRNSSRHIIIRTPNALNKDRILKAVREKGQVTYKGKPI     297
Rat             NLKKEIPIGIQEAYRTPNRLDQKRNTSRHIIVKTPNAQNKERILKAVREKGQVTYKGRPI     269
```

```
              ** ::  : ***  ***:* ..:* : ****::  :.  *:::*:*.****:** **:**

Human         RLTADLSAETLQARREWGPIFNILKEKNFQPRISYPAKLSFISEGEIKYFIDKQMLRDFV      307
Mouse         RITPDFSPETMKARRAWTDVIQTLREHKCQPRLLYPAKLSITIDGETKVFHDKTKFTHYL      357
Rat           RITPDFSPETMKARRSWTDVIQTLREHKCQPRLLYPAKLSINIDGETKIFHDKTKFTQYL      329
              *:* *:* **::*** *  ::: *:*:: ***: ******:  :** * * **  : .::

Human         TTRPALKELLKEALNMERNNRYQPLQNHAKM 338
Mouse         STNPALQRIITEKKQYKDGNHALEKTR--R- 385
Rat           STNPALQRIINGKAQHKEASYTLEEAR--N- 357
              :*.***:.::.   : :  .       .  .
```

>Human L1 ORF2

MTGSTSHITILTLNINGLNSAIKRHRLASWIKSQDPSVCCIQETHLTCRDTHRLKIKGWRKIYQANGKQKKAGVAILVSDK
TDFKPTKIKRDKEGHYIMVKGSIQQEELTILNIYAPNTGAPRFIKQVLSDLQRDLDSHTLIMGDFNTPLSTLDRSTRQKVN
KDTQELNSALHQADLIDIYRTLHPKSTEYTFFSAPHHTYSKIDHIVGSKALLSKCKRTEIITNYLSDHSAIKLELRIKNLTQSRS
TTWKLNNLLLNDYWVHNEMKAEIKMFFETNENKDTTYQNLWDAFKAVCRGKFIALNAYKRKQERSKIDTLTSQLKELE
KQEQTHSKASRRQEITKIRAELKEIETQKTLQKINESRSWFFERINKIDRPLARLIKKKREKNQIDTIKNDKGDITTDPTEIQT
TIREYYKHLYANKLENLEEMDTFLDTYTLPRLNQEEVESLNRPITGSEIVAIINSLPTKKSPGPDGFTAEFYQRYKEELVPFLL
KLFQSIEKEGILPNSFYEASIILIPKPGRDTTKKENFRPISLMNIDAKILNKILANRIQQHIKKLIHHDQVGFIPGMQGWFNIR
KSINVIQHINRAKDKNHMIISIDAEKAFDKIQQPFMLKTLNKLGIDGTYFKIIRAIYDKPTANIILNGQKLEAFPLKTGTRQG
CPLSPLLFNIVLEVLARAIRQEKEIKGIQLGKEEVKLSLFADDMIVYLENPIVSAQNLLKLISNFSKVSGYKINVQKSQAFLYT
NNRQTESQIMGELPFTIASKRIKYLGIQLTRDVKDLFKENYKPLLKEIKEETNKWKNIPCSWVGRINIVKMAILPKVIYRFN
AIPIKLPMTFFTELEKTTLKFIWNQKRARIAKSILSQKNKAGGITLPDFKLYYKATVTKTAWYWYQNRDIDQWNRTEPSEI
MPHIYNYLIFDKPEKNKQWGKDSLFNKWCWENWLAICRKLKLDPFLTPYTKINSRWIKDLNVKPKTIKTLEENLGITIQDI
GVGKDFMSKTPKAMATKDKIDKWDLIKLKSFCTAKETTIRVNRQPTTWEKIFATYSSDKGLISRIYNELKQIYKKKTNNPIK
KWAKDMNRHFSKEDIYAAKKHMKKCSSSLAIREMQIKTTMRYHLTPVRMAIIKKSGNNRCWRGCGEIGTLLHCWWD
CKLVQPLWKSVWRFLRDLELEIPFDPAIPLLGIYPNEYKSCCYKDTCTRMFIAALFTIAKTWNQPKCPTMIDWIKKMWHI
YTMEYYAAIKNDEFISFVGTWMKLETIILSKLSQEQKTKHRIFSLIGGN

>Mice L1ORF2

MPTLTTKIKGSNNYFSLISLNINGLNSPIKRHRLTDWLHKQDPTFCCLQETHLREKDRHYLRVKGWKTTFQANGLKKQA
GVAILISDKIDFQPKVIKKDKEGHFILIKGKILQEELSILNIYAPNARAATFIRDTLVKLKAYIAPHTIIVGDFNTPLSSKDRSWK
QKLNRDTVKLTEVMKQMDLTDIYRAFYPKTKGYTFFSAPHGTFSKIDHIIGHKTGLNRYKNIEIVPCILSDHHGLRLIFNDN
INNGKPTFTWKLNNTLFNDTLVKEGIKKEIKDFLEFNENEATTYPNLWDTMKAFLRGKLIALSASKKKRETAHTSSLTTHL
KALEKKEAHSPKRSRRQEIIKLRGEINQVETRRTIQRINQTRSWFFEKINKIDKPLARLTKGHRDKILINKIRNEKGDITTDPE
EIQNTIRSFYTRLYSTKLENLDEMDKFLDRYQVPKLNQDQVDHLNSPISPKEIEAVINSLPTKKSPGPDGFSAEFYQTFKED
LIPILHKLFHKIEVEGTLPNSFYEATITLIPKPQKDPTKIENFRPISLMNIDAKILNKILANRIQEHIKAIIHPDQVGFIPGMQG
WFNIRKSINVIHYINKLKDKNHMIISLDAEKAFDKIQHPFMIKVLERSGIQGPYLNMIKAIYSKPVANIKVNGEKLEAIPLKS
GTRQGCPLSPYLFNIVLEVLARAIRQQKEIKGIQIGKEEVKISLFADDMIVYISDPKNSTRELINLINSFGEVAGYKINSNKS
MAFLYTKNKQAEKEIRETTPFSIVTNNIKYLGVTLTKEVKDLYDKNFKSLKKEIKEDLRRWKDLPCSWIGRINIVKMAILPK
AIYRFNAIPIKIPTQFFNELEGAICKFVWNNKKPRIAKSLLKDKRTSGGITMPDLKLYYRAIVIKTAWYWYRDRQVDQWN
RIEDPEMNPHTYGHLIFDKGAKTIQWKKDSIFNNWCWHNWLLSCRRMRIDPYLSPCTKVKSKWIKELHIKPETLKLIEEK
VGKSLEDMGTGEKFLNRTAMACAVRSRIDKWDLMKLQSFCKAKDTVNKTKRPPTDWERIFTYPKSDRGLISNIYKELKK
VDFRKSNNPIKKWGSELNKEFSPEEYRMAEKHLKKCSTSLIIREMQIKTTLRFHLTPVRMAKIKNSGDSRCWRGCGERGT
LLHCWWECRLVQPLWKSVWRFLRKLDIVLPEDPAIPLLGIYPEDAPTGKKDTCSTMFIAALFIIARSWKEPRCPSTEEWI
QKMWYIYTMEYYSAIKKNEFMKFLAKWMDLEGIILSEVTHSQRNSHNMYSLISGY

>Rat L1ORF2

MNIKGNNNHYSLISLNINGLNSPIKRHRLTNWIRNEDPAFCCLQETHLRDKDRHYLRVKGWKTTFQANGQKKQAGVAI
LISNKINFQLKVIKKDKEGHFIFIKGKIHQDELSILNIYAPNTRAPTYVKETLLKLKTHIAPHTIIVGDFNTPLSSMDRSWKQK
LNSDVDRLREVMSQMDLTDIYRTFYPKAKGYTFFSAPHGTFSKIDHIIGQKTGLNRYRKIEIIPCVLSDHHGLKLVFNNNK
GRMPTYTWKLNNALLNDNLVKEEIKKEIKNFLEFNENENTTYSNLWDTMKAVLRGKLIALSACRKKQERAYVSSMTAHL
KALEQKEANTPRRSRRQEIIKLRAEINQVETKRTIERINRTKSWFFEKINKIDKPLARLTRGHRECVQINKIRNEKGDITTDS
EEIQKIIRSYYKNLYSTKFENLQEMDYFLDRYQVSKLNQEQINQLNNPITPKEIEAVIKGLPTKKSPGPDGFSAEFYQTFIED
LIPILSKLFHKIETDGALPNSFYESTITLIPKPHKDTTKKGNFRPISLMNIDAKILNKILANRIQEHIKTIIHHDQVGFIPGMQG
WFNIRKTINVIHYINKLKEQNHMIISLDAEKAFDKIQHPFMIKVLERIGIQGPYLNIVKAIYSKPVANIKLNGEKLEAIPLKSG
TRQGCPLSPYLFNIVLEVLARAIRQQKEIKGMQIGKEEVKISLFADDMIVYLSDPKSSTRELLKLINNFSKVAGYKINSNKSV
AFLYTKEKQAEKEIRETTPFIIDPNNIKYLGVALTKQVKDLYNKNFKTLKKEIEEDLRRWKDLPCSWIGRINIVKMAILPKAI
YRFNAIPIKIPIQFFKELDRTICKFIWNNKKPRIAKAILNNKRTSGGITIPELKQYYRAIVIKTAWYWYRDRQIDQWNRIEDP
EMNPHTYGHLIFDKGAKTIQWKKDSIFSKWCWFNWRATCRRMQIDPCLSPCTKLKSKWIKDLHIKPDTLKLIEEKLGKH
LEHMGTGKNFLNKTPMAYALRSRIDKWDLIKLQSFCKAKDTVVRTKRQPTDWEKIFTNPTTDRGLISKIYKELKKLDRRE
TNNPIKKWGSELNKEFTAEECRMAEKHLKKCSTSLVIREMQIKTTLRFHLTPVRLAKIKNSGDSRCWRGCGERGTLLHC
WWDCRLVKPFWKSVWRFLRKLDIELPEDPAIPLLGIYPKDASTYKRDTCSTMFIAALFIIARKWKEPRCPSTEEWIQKM
WYIYTMEYYSAIKNNKFMKFVGKWLELENIILSELTQSQKDIHGMHSLISGY

CLUSTAL multiple sequence alignment of Human, Mice and Rat L1ORF2 protein sequences
Yellow shaded part from human L1ORF2 was used to make L1ORf2 specific antibody

```
Human_ORF2      -------MTGSTSHITILTLNINGLNSAIKRHRLASWIKSQDPSVCCIQETHLTCRDTHR 53
ORF2_GF21       MPTLTTKITGSNNYLSLISLNINGLNSPIKRHRLTDWLHKQDPTFCCLQETHLREKDRHY 60
RAT_ORF2        -----MNIKGNNNHYSLISLNINGLNSPIKRHRLTNWIRNEDPAFCCLQETHLRDKDRHY 55
                       :.*...: ::::******** ******:.*::.:**:.**:*****   :* *

Human_ORF2      LKIKGWRKIYQANGKQKKAGVAILVSDKTDFKPTKIKRDKEGHYIMVKGSIQQEELTILN 113
ORF2_GF21       LRVKGWKTIFQANGLKKQAGVAILISDKIDFQPKVIKKDKEGHFILIKGKILQEELSILN 120
RAT_ORF2        LRVKGWKTTFQANGQKKQAGVAILISNKINFQLKVIKKDKEGHFIFIKGKIHQDELSILN 115
                *::***:. :**** :*:******:*:* :*: . **:*****:*::**.* *:**:***

Human_ORF2      IYAPNTGAPRFIKQVLSDLQRDLDSHTLIMGDFNTPLSTLDRSTRQKVNKDTQELNSALH 173
ORF2_GF21       IYAPNARAATFIKDTLVKLKAHIAPHTIIVGDLNTPLSSMDRSWKQKLNRDTVKLTEVMK 180
RAT_ORF2        IYAPNTRAPTYVKETLLKLKTHIAPHTIIVGDFNTPLSSMDRSWKQKLNSDVDRLREVMS 175
                *****: *  ::*:.* .*: .:  **:*:**:*****::*** :**:* *. .* ..:

Human_ORF2      QADLIDIYRTLHPKSTEYTFFSAPHHTYSKIDHIVGSKALLSKCKRTEIITNYLSDHSAI 233
ORF2_GF21       QMDLTDIYRIFNPKTKGYTFFSAPHGTFSKIDHIIGHKTGLNRYKNIEIVPCILSDHHGL 240
RAT_ORF2        QMDLTDIYRTFYPKAKGYTFFSAPHGTFSKIDHIIGQKTGLNRYRKIEIIPCVLSDHHGL 235
                * ** **** : **:. ******** *:******:* *: *.: :. **:    **** .:

Human_ORF2      KLELRIKNLTQSRSTTWKLNNLLLNDYWVHNEMKAEIKMFFETNENKDTTYQNLWDAFKA 293
ORF2_GF21       RLIFNNNIKNGKPTFTWKLNNTLLNDTLVKEGIKKEIKDFLEFNENEATTYPNLWDTMKA 300
RAT_ORF2        KLVFNNN-KGRMPTYTWKLNNALLNDNLVKEEIKKEIKNFLEFNENENTTYSNLWDTMKA 294
                :* :. :      : ****** ****  *:: :* *** *:* ***: *** ****::**

Human_ORF2      VCRGKFIALNAYKRKQERSKIDTLTSQLKELEKQEQTHSKASRRQEITKIRAELKEIETQ 353
ORF2_GF21       FLRGKLIALSTSKKKRERAHTSSLTTHLKALEKKEANSPKRSRRQEIIKLRGEINQVETR 360
RAT_ORF2        VLRGKLIALSACRKKQERAYVSSMTAHLKALEQKEANTPRRSRRQEIIKLRAEINQVETK 354
                . ***:***.: ::*:**:  .::*::** **::* .   : ****** *:*.*::::**:

Human_ORF2      KTLQKINESRSWFFERINKIDRPLARLIKKKREKNQIDTIKNDKGDITTDPTEIQTTIRE 413
ORF2_GF21       RTIQRINQTRSWFFEKINKIDKHLARLTRGQRDKILINKIRNEKGDITTDPEEIQNTIRS 420
RAT_ORF2        RTIERINRTKSWFFEKINKIDKPLARLTRGHRECVQINKIRNEKGDITTDSEEIQKIIRS 414
                :*:::**.::*****:*****: **** : :*:   *:.*:*:*******  ***. **.
```

```
Human_ORF2      YYKHLYANKLENLEEMDTFLDTYTLPRLNQEEVESLNRPITGSEIVAIINSLPTKKSPGP 473
ORF2_GF21       FYKSLYSTKLENLDEMDKFLDKYQVPKLNQDQVDLLNSPISPKEIEAVINSLPAKKSPGP 480
RAT_ORF2        YYKNLYSTKFENLQEMDYFLDRYQVSKLNQEQINQLNNPITPKEIEAVIKGLPTKKSPGP 474
                :** **:.*:***:*** *** * : :***:::: ** **: .** *:*:.**:******

Human_ORF2      DGFTAEFYQRYKEELVPFLLKLFQSIEKEGILPNSFYEASIILIPKPGRDTTKKENFRPI 533
ORF2_GF21       DGFSAEFYQTFKEDLIPVLHKLFHRIEVEGTLPNSFYEATITLIPKPQKDPTKIENFRPI 540
RAT_ORF2        DGFSAEFYQTFIEDLIPILSKLFHKIETDGALPNSFYESTITLIPKPHKDTTKKGNFRPI 534
                ***:***** : *:*:*.* ***: ** :* *******::* ***** :* **  *****

Human_ORF2      SLMNIDAKILNKILANRIQQHIKKLIHHDQVGFIPGMQGWFNIRKSINVIQHINRAKDKN 593
ORF2_GF21       SLMNIDAKILNKILANRIQEHIKEIIHPDQVGFIPGMQGWFNIRKSINVIHYINKLKDKN 600
RAT_ORF2        SLMNIDAKILNKILANRIQEHIKTIIHHDQVGFIPGMQGWFNIRKTINVIHYINKLKEQN 594
                *******************:*** :** *****************:****::**: *::*

Human_ORF2      HMIISIDAEKAFDKIQQPFMLKTLNKLGIDGTYFKIIRAIYDKPTANIILNGQKLEAFPL 653
ORF2_GF21       HMIISLDAEKAFDKIQHPFMIKVLERSGIQGPYLNIIKAIYSKPVANIKVNGEKLEAIPL 660
RAT_ORF2        HMIISLDAEKAFDKIQHPFMIKVLERIGIQGPYLNIVKAIYSKPVANIKLNGEKLEAIPL 654
                *****:**********:***:*.*:: **:* *::*::***.**.*** :**:****:**

Human_ORF2      KTGTRQGCPLSPLLFNIVLEVLARAIRQEKEIKGIQLGKEEVKLSLFADDMIVYLENPIV 713
ORF2_GF21       KSGTRQGCPLSPYLFNIVLEVLARAIRQQKEIKGIQIGKEEVKISLFADDMIVYISDPKN 720
RAT_ORF2        KSGTRQGCPLSPYLFNIVLEVLARAIRQQKEIKGMQIGKEEVKISLFADDMIVYLSDPKS 714
                *:********** ***************:*****:*:******:**********:.:*  

Human_ORF2      SAQNLLKLISNFSKVSGYKINVQKSQAFLYTNNRQTESQIMGELPFTIASKRIKYLGIQL 773
ORF2_GF21       STRELLNLINSFGEVAGYKINSNKSMAFLYTKNKQAEKEIRETTPFSIVTNNIKYLGVTL 780
RAT_ORF2        STRELLKLINNFSKVAGYKINSNKSVAFLYTKEKQAEKEIRETTPFIIDPNNIKYLGVAL 774
                *:::**:**..*.:*:***** :** *****:::*:*.:*    ** *  :.*****: *

Human_ORF2      TRDVKDLFKENYKPLLKEIKEETNKWKNIPCSWVGRINIVKMAILPKVIYRFNAIPIKLP 833
ORF2_GF21       TKEVKDLYDKNFKSLKKEIKEDLRRWKDLPCSWIGRINIVKMAILPKAIYRFNAIPIKIP 840
RAT_ORF2        TKQVKDLYNKNFKTLKKEIEEDLRRWKDLPCSWIGRINIVKMAILPKAIYRFNAIPIKIP 834
                *::****:.:*:* * ***:*: .:**::****:*************.**********:*

Human_ORF2      MTFFTELEKTTLKFIWNQKRARIAKSILSQKNKAGGITLPDFKLYYKATVTKTAWYWYQN 893
ORF2_GF21       TQFFNELEGAICKFIWNNKKPRIAKTLLKDKRTSGGITMPDLKLYYRAIVIKTAWYWYRD 900
RAT_ORF2        IQFFKELDRTICKFIWNNKKPRIAKAILNNKRTSGGITIPELKQYYRAIVIKTAWYWYRD 894
                  **.**: :  *****:*: ****::*.:*..:****:*::* **:* * *******::

Human_ORF2      RDIDQWNRTEPSEIMPHIYNYLIFDKPEKNKQWGKDSLFNKWCWENWLAICRKLKLDPFL 953
ORF2_GF21       RQVDQWNRIEDPEMNPHTYGHLIFDKGAKTIQWKKDSIFNNWCWHNWLLSCRRMRIYPYL 960
RAT_ORF2        RQIDQWNRIEDPEMNPHTYGHLIFDKGAKTIQWKKDSIFSKWCWFNWRATCRRMQIDPCL 954
                *::***** *  *: ** *.:*****  *. ** ***:*.:*** **   **:::: * *

Human_ORF2      TPYTKINSRWIKDLNVKPKTIKTLEENLGITIQDIGVGKDFMSKTPKAMATKDKIDKWDL 1013
ORF2_GF21       SPCTKVKSKWIKELHIKPETLKLIEEKVGKSLEDMGTGEKFLNRTALACSVRSRIDKWDL 1020
RAT_ORF2        SPCTKLKSKWIKDLHIKPDTLKLIEEKLGKHLEHMGTGKNFLNKTPMAYALRSRIDKWDL 1014
                :* **::*:***:*::**.*:* :**::*  ::.:*.*:.*:.:*  * : :.:******

Human_ORF2      IKLKSFCTAKETTIRVNRQPTTWEKIFATYSSDKGLISRIYNELKQIYKKKTNNPIKKWA 1073
ORF2_GF21       MKLQSFCKAKDTVNKTKRPPTDWERIFTYPKSDRGLISNIYKELKKVDFRKSNNPIKKWG 1080
RAT_ORF2        IKLQSFCKAKDTVVRTKRQPTDWEKIFTNPTTDRGLISKIYKELKKLDRRETNNPIKKWG 1074
                :**:***.**:*. :.:* ** **:**:  .:*:****.**:***::  :::*******.

Human_ORF2      KDMNRHFSKEDIYAAKKHMKKCSSSLAIREMQIKTTMRYHLTPVRMAIIKKSGNNRCWRG 1133
ORF2_GF21       SELNKEFSPEEYRMAEKHLKKCSTSLIIREMQIKTTLRFHLTPVRMAKIKNSGDSRCWRG 1140
RAT_ORF2        SELNKEFTAEECRMAEKHLKKCSTSLVIREMQIKTTLRFHLTPVRLAKIKNSGDSRCWRG 1134
                .::*:.*: *:   *:**:****:** *********:*:******:* **:**:.*****

Human_ORF2      CGEIGTLLHCWWDCKLVQPLWKSVWRFLRDLELEIPFDPAIPLLGIYPNEYKSCCYKDTC 1193
ORF2_GF21       CGERGTLLHCWWDCRLVQPLWKSVWRFLRKLDIVLPEDPAIPLLGIYPEDAP-TGKKDTC 1199
RAT_ORF2        CGERGTLLHCWWDCRLVKPFWKSVWRFLRKLDIELPEDPAIPLLGIYPKDAS-TYKRDTC 1193
                *** **********:**:*:*********.*:: :* ***********::      :***

Human_ORF2      TRMFIAALFTIAKTWNQPKCPTMIDWIKKMWHIYTMEYYAAIKNDEFISFVGTWMKLETI 1253
ORF2_GF21       STMFIAALFIIARSWKEPRCPSTEEWIQKMWYIYTMEYYSAIKKNEFMKFLAKWMDLEGI 1259
RAT_ORF2        STMFIAALFIIARKWKEPRCPSTEEWIQKMWYIYTMEYYSAIKNNKFMKFVGKWLELENI 1253
                : ******* **:.*::*:**:  :**:***:*******:***:::*:.*:..*:.** *
```

```
Human_ORF2      ILSKLSQEQKTKHRIFSLIGGN 1275
ORF2_GF21       ILSEVTHSQRNSHNMYSLISGY 1281
RAT_ORF2        ILSELTQSQKDIHGMHSLISGY 1275
                ***::::.*:  * :.***.*
```

>hL1ORF2RT
EFYQRYKEELVPFLLKLFQSIEKEGILPNSFYEASIILIPKPGRDTTKKENFRPISLMNIDAKILNKILANRIQQHIKKL
>mL1ORF2RT

EFYQTFKEDLIPVLHKLFHRIEVEGTLPNSFYEATITLIPKPQKDPTKIENFRPISLMNIDAKILNKILANRIQEHIKEI
>rL1ORF2RT

EFYQTFIEDLIPILSKLFHKIETDGALPNSFYESTITLIPKPHKDTTKKGNFRPISLMNIDAKILNKILANRIQEHIKTI

```
CLUSTAL(1.2.4) multiple sequence alignment


hL1ORF2RT      EFYQRYKEELVPFLLKLFQSIEKEGILPNSFYEASIILIPKPGRDTTKKENFRPISLMNI  60
mL1ORF2RT      EFYQTFKEDLIPVLHKLFHRIEVEGTLPNSFYEATITLIPKPQKDPTKIENFRPISLMNI  60
rL1ORF2RT      EFYQTFIEDLIPILSKLFHKIETDGALPNSFYESTITLIPKPHKDTTKKGNFRPISLMNI  60
               **** : *:*:*.* ***: ** :* *******::* ***** :* **  **********

hL1ORF2RT      DAKILNKILANRIQQHIKKL        80
mL1ORF2RT      DAKILNKILANRIQEHIKEI        80
rL1ORF2RT      DAKILNKILANRIQEHIKTI        80
               **************:*** :
```

```
Percent Identity  Matrix - created by Clustal2.1
     1: hL1ORF2RT    100.00    76.25    73.75
     2: mL1ORF2RT     76.25   100.00    83.75
     3: rL1ORF2RT     73.75    83.75   100.00
```

Original Gels and Immunoblots shown in main figures attached :

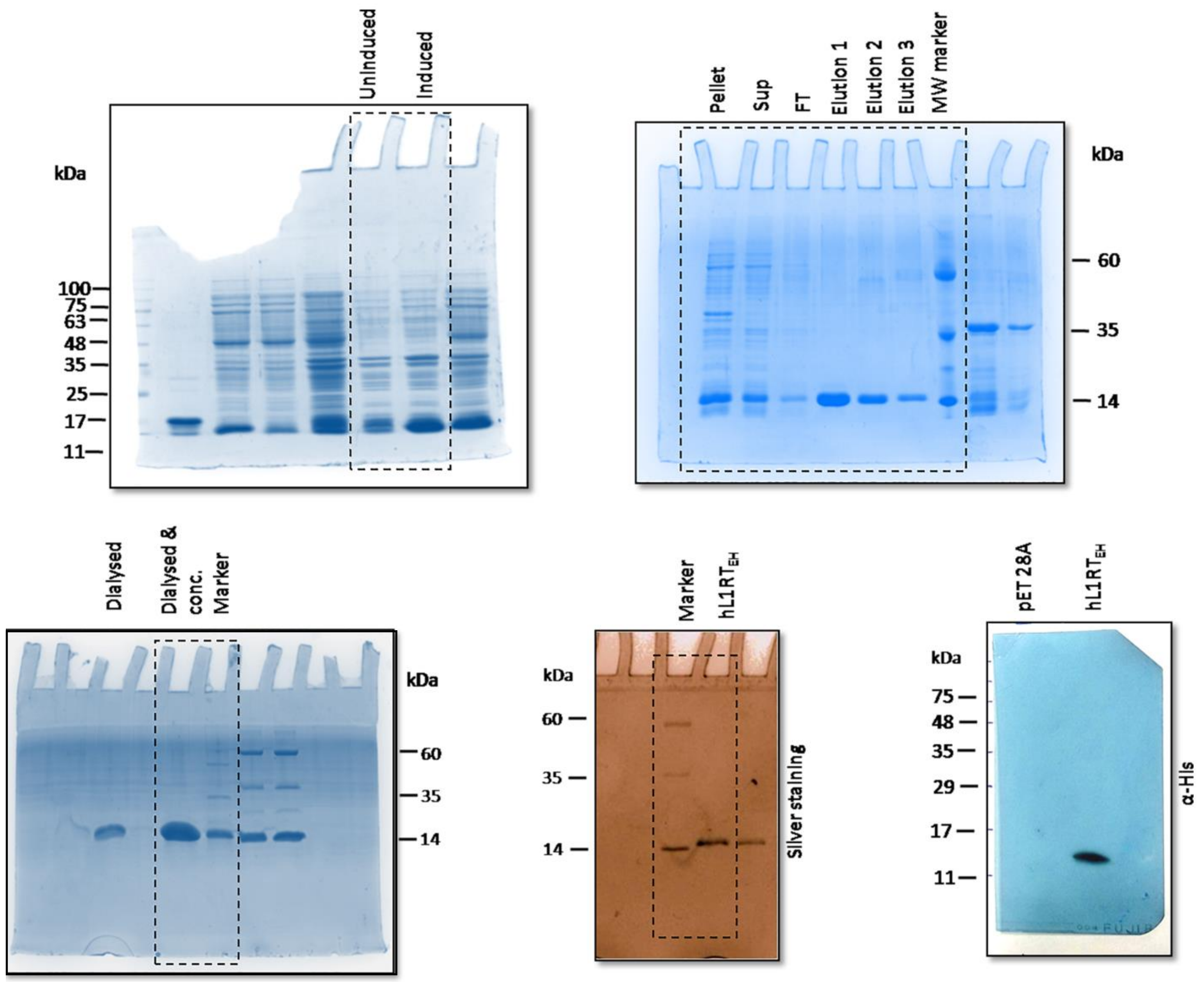

Original SDS Page and western blots of Main Figure 1

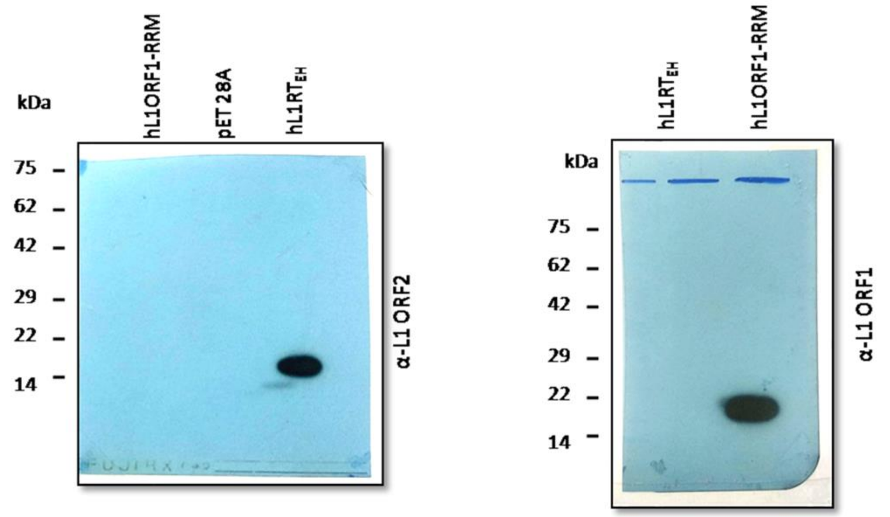

Original western blots of Main Figure 2 (A)

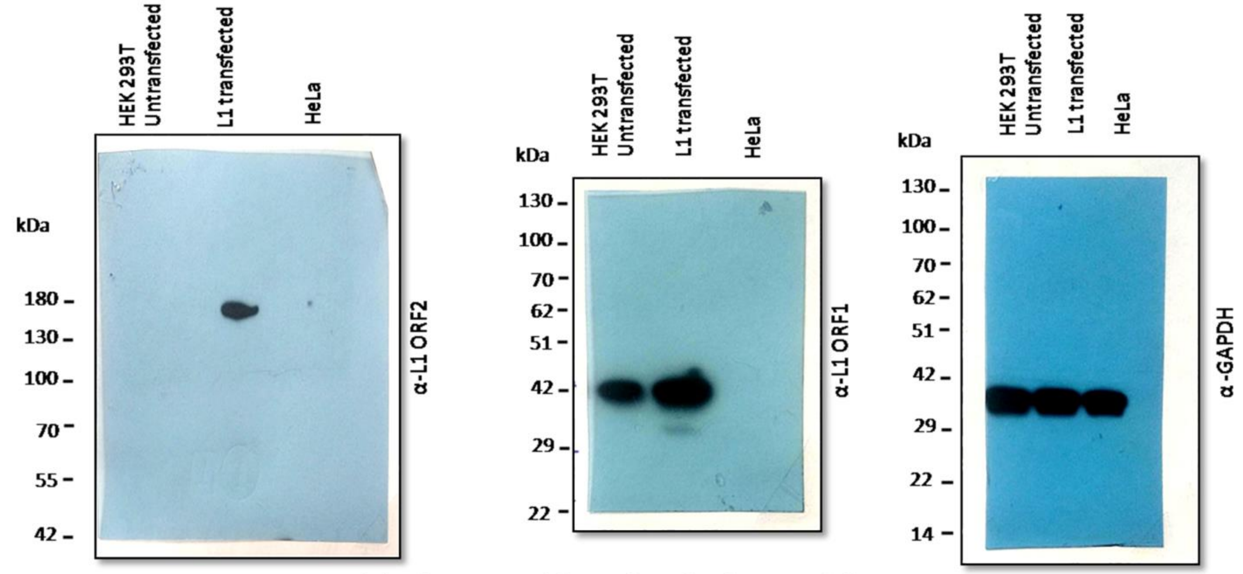

Original western blots of Main Figure 2 (B)

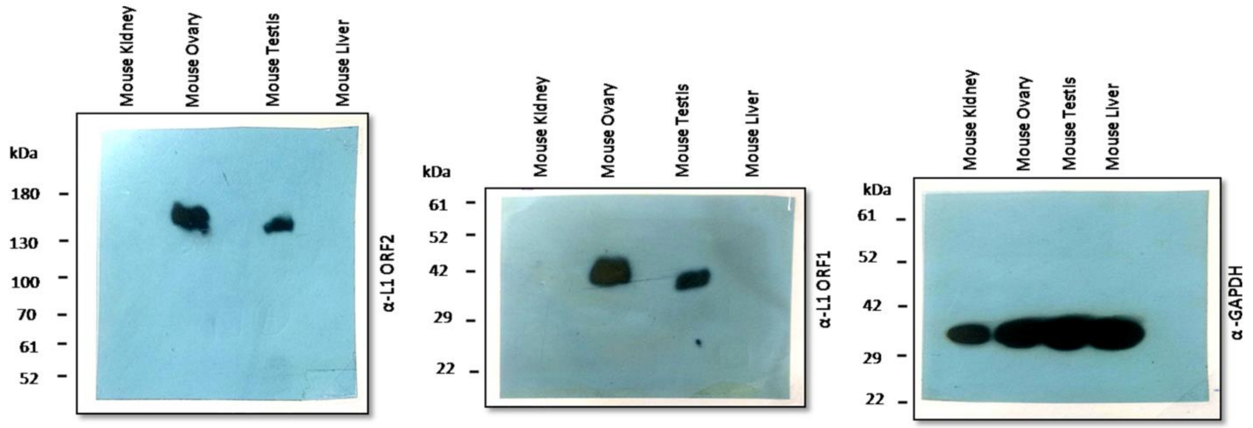

Original western blots of Mouse tissues of Main Figure 3 (A)

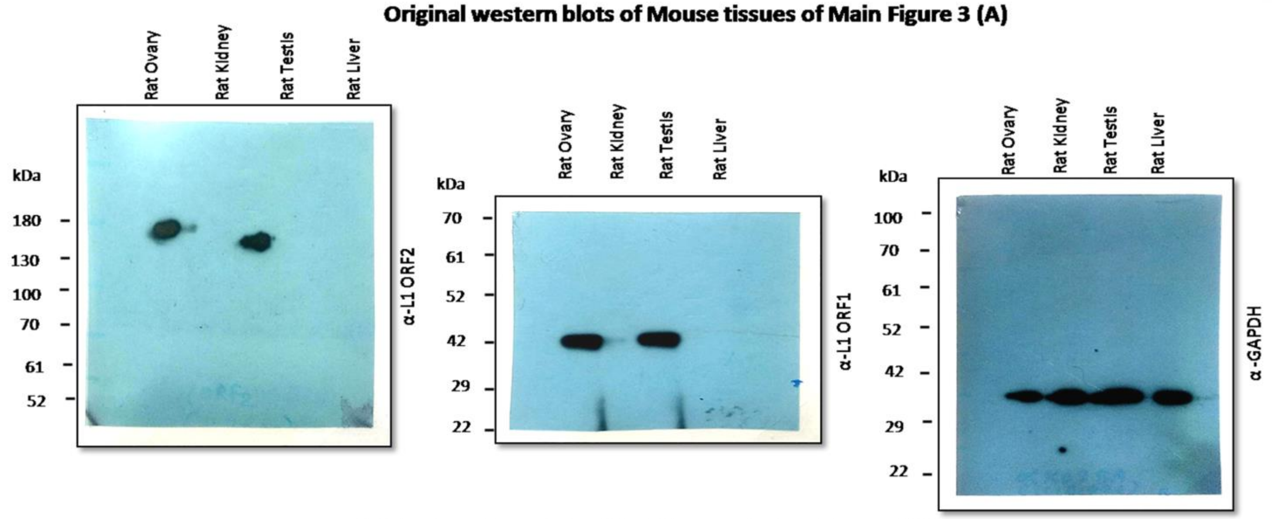

Original western blots of Rat tissues of Main Figure 3 (B)

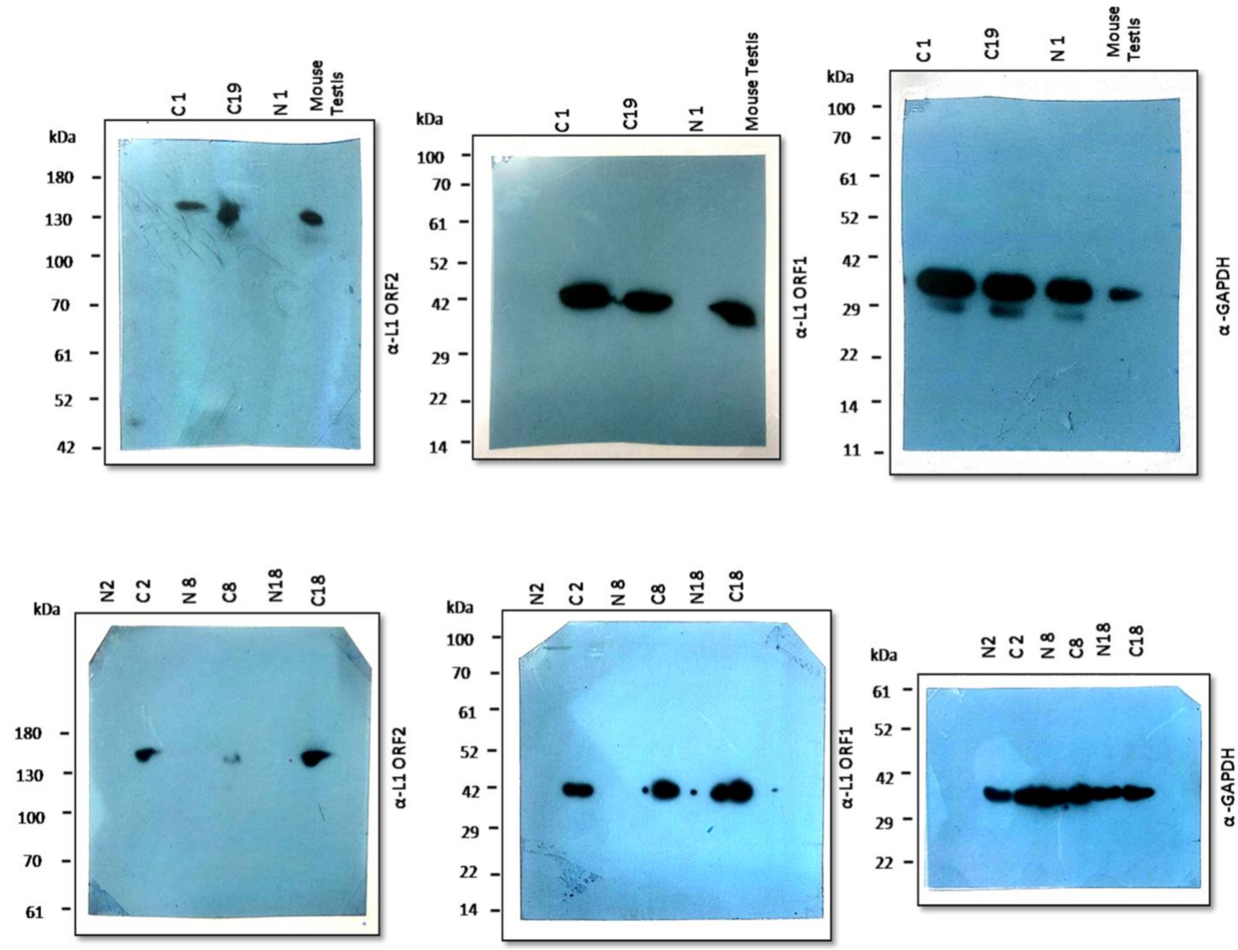

Original western blots of Main Figure 6 (B)

Original Gels and Immunoblots shown in supplementary figures attached :

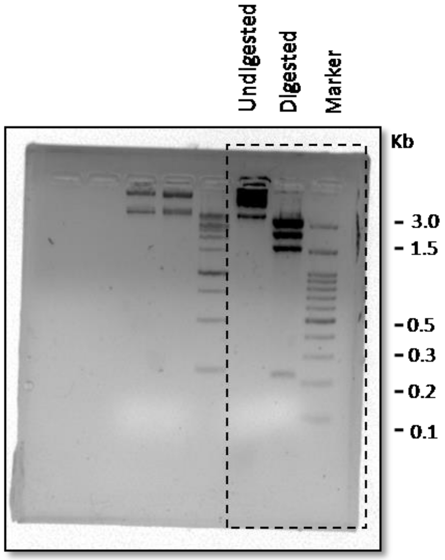

JCC5 digested with
EcoRI and *HindIII*

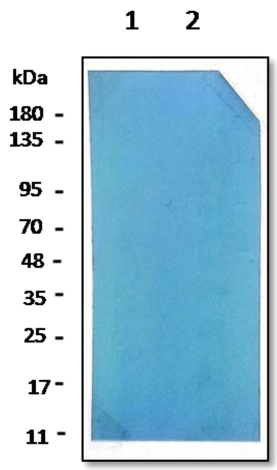

Western blot with
Mouse pre-bleed

1. E. coli total lysate, induced pETL1RT$_{EH}$
2. HEK293T total lysate (L1RPEGFP transfected)

Original Agarose gel and western blot of Supplementary Figure 1

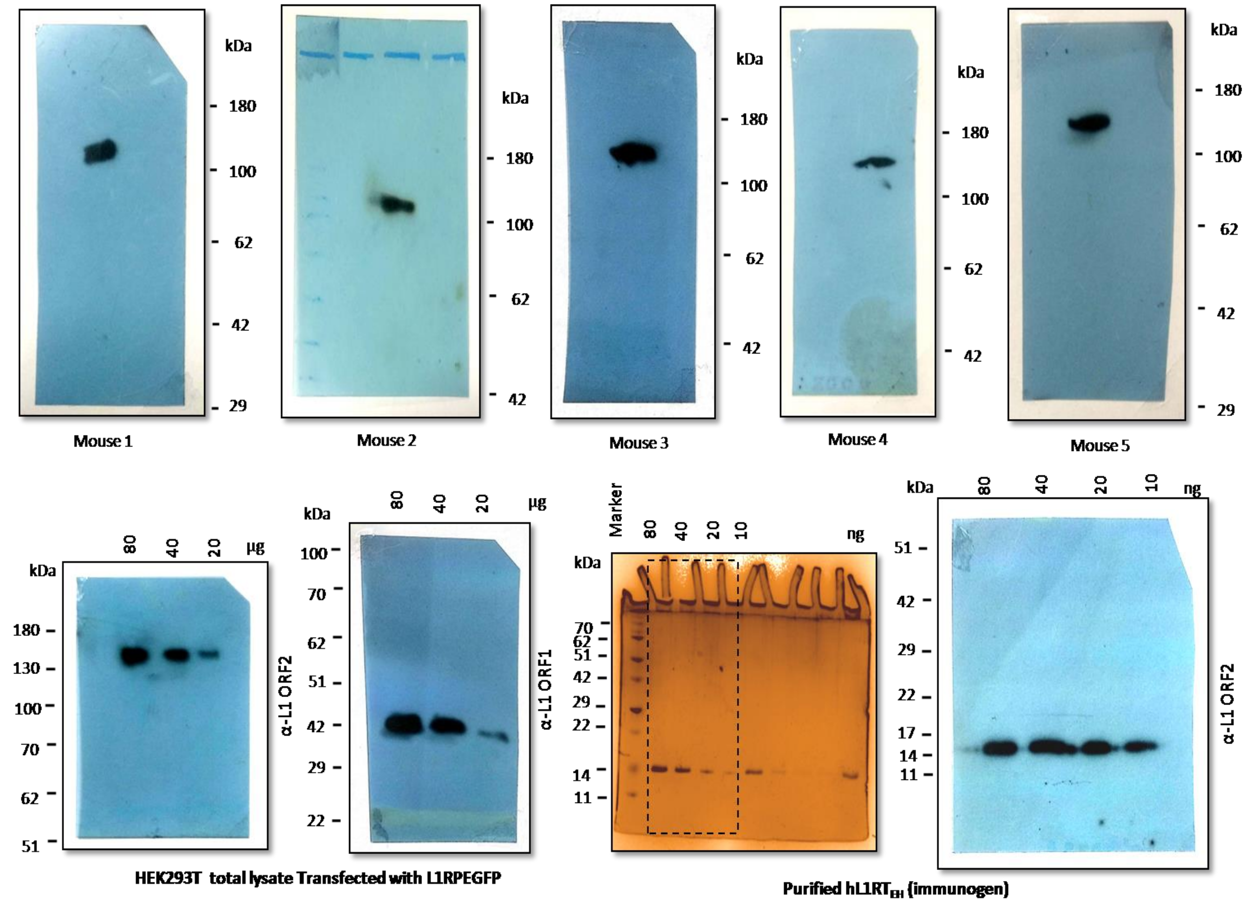

Original gel and western blots of Supplementary Figure 2

Original Hand written hard copies of Immunoblots shown in main figures attached :

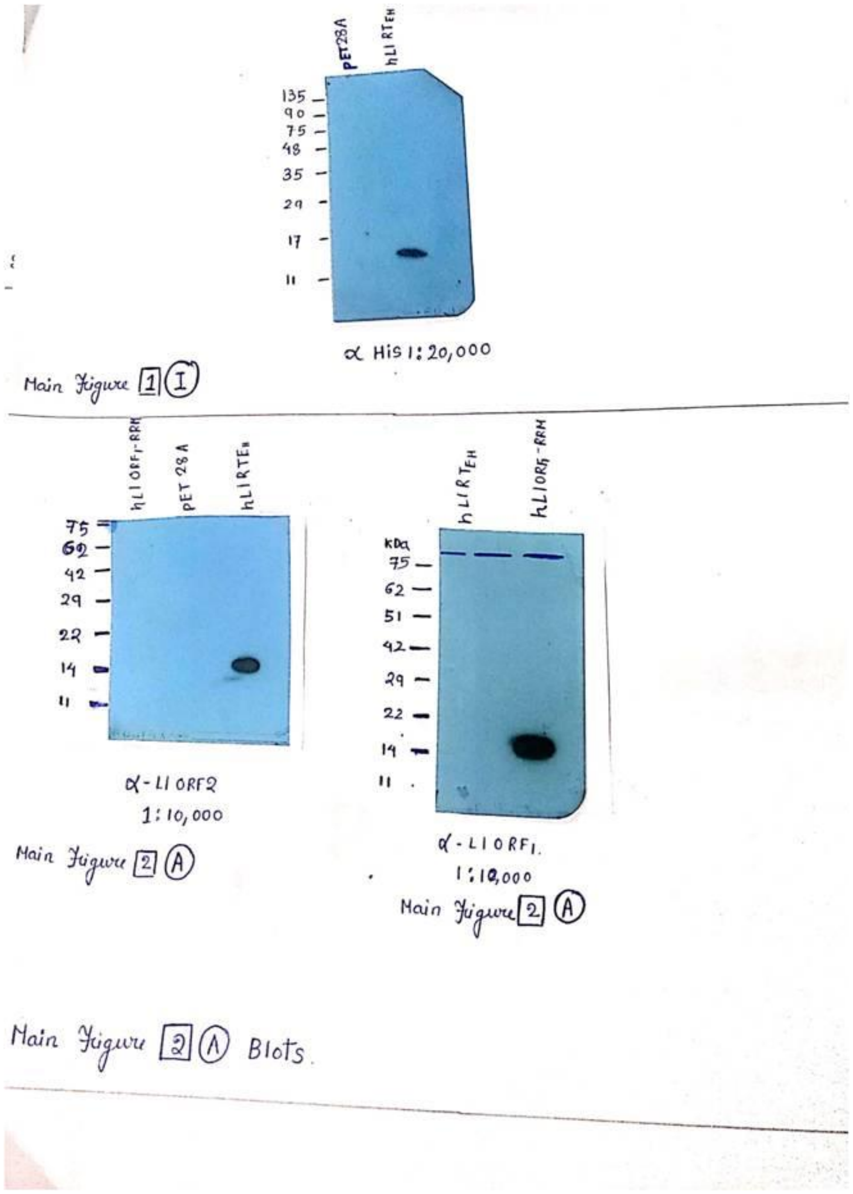

Page 1

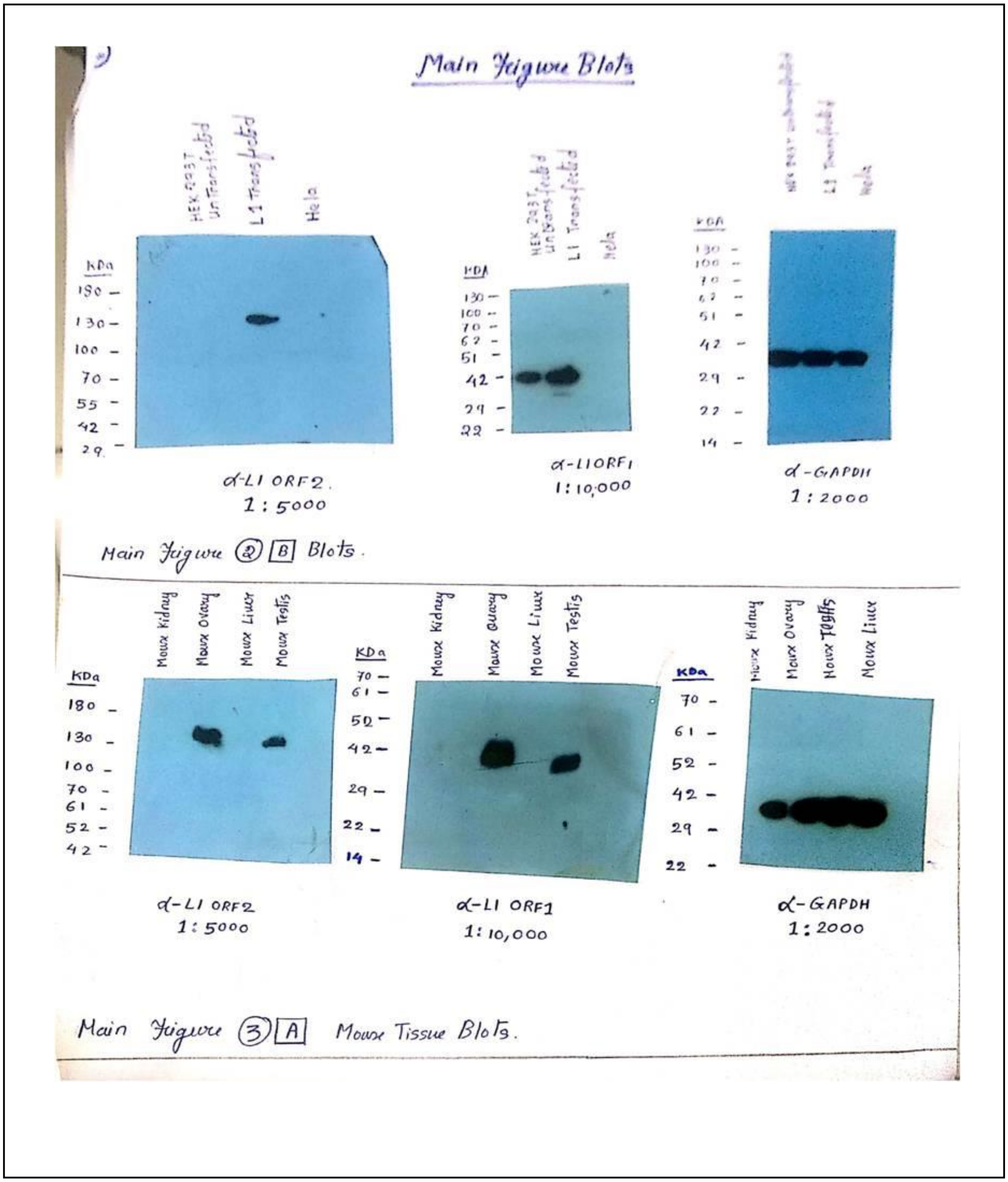
Main Figure Blots
α-L1 ORF2.
1:5000
α-L1ORF1
1:10,000
α-GAPDH
1:2000
Main Figure ② B Blots.
α-L1 ORF2
1:5000
α-L1 ORF1
1:10,000
α-GAPDH
1:2000
Main Figure ③ A Mouse Tissue Blots.

Page 2

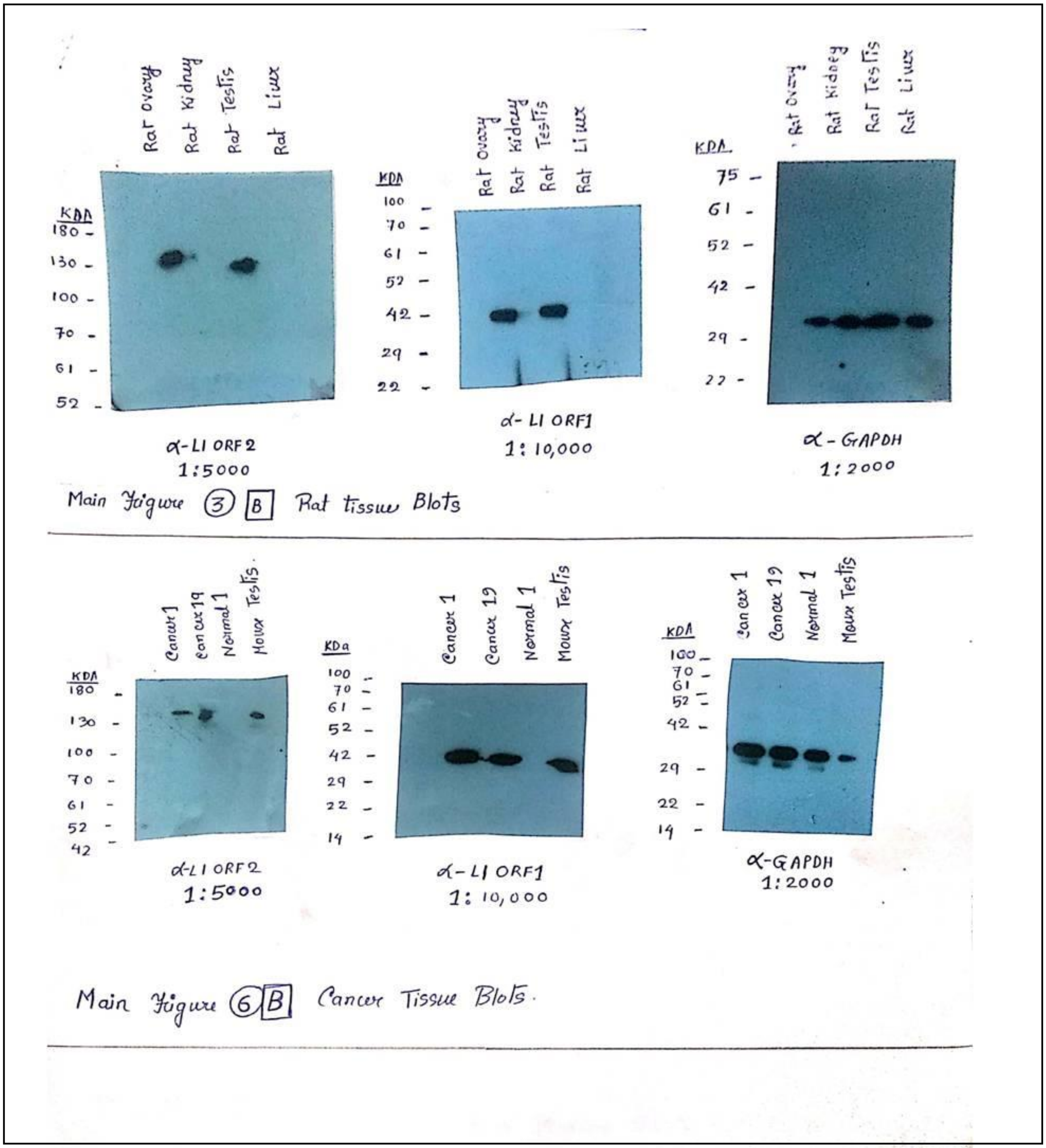
Rat ovary
Rat Kidney
Rat Testis
Rat Liver
KDA
180
130
100
70
61
52
α-L1 ORF2
1:5000
KDA
100
70
61
52
42
29
22
α- L1 ORF1
1: 10,000
KDA
75
61
52
42
29
22
α - GAPDH
1: 2000
Main Figure 3 B Rat tissue Blots
Cancer 1
Cancer 19
Normal 1
Mouse Testis
KDA
180
130
100
70
61
52
42
α-L1 ORF2
1:5000
KDa
100
70
61
52
42
29
22
14
α- L1 ORF1
1: 10,000
KDA
100
70
61
52
42
29
22
14
α-GAPDH
1: 2000
Main Figure 6 B Cancer Tissue Blots.

Page 3

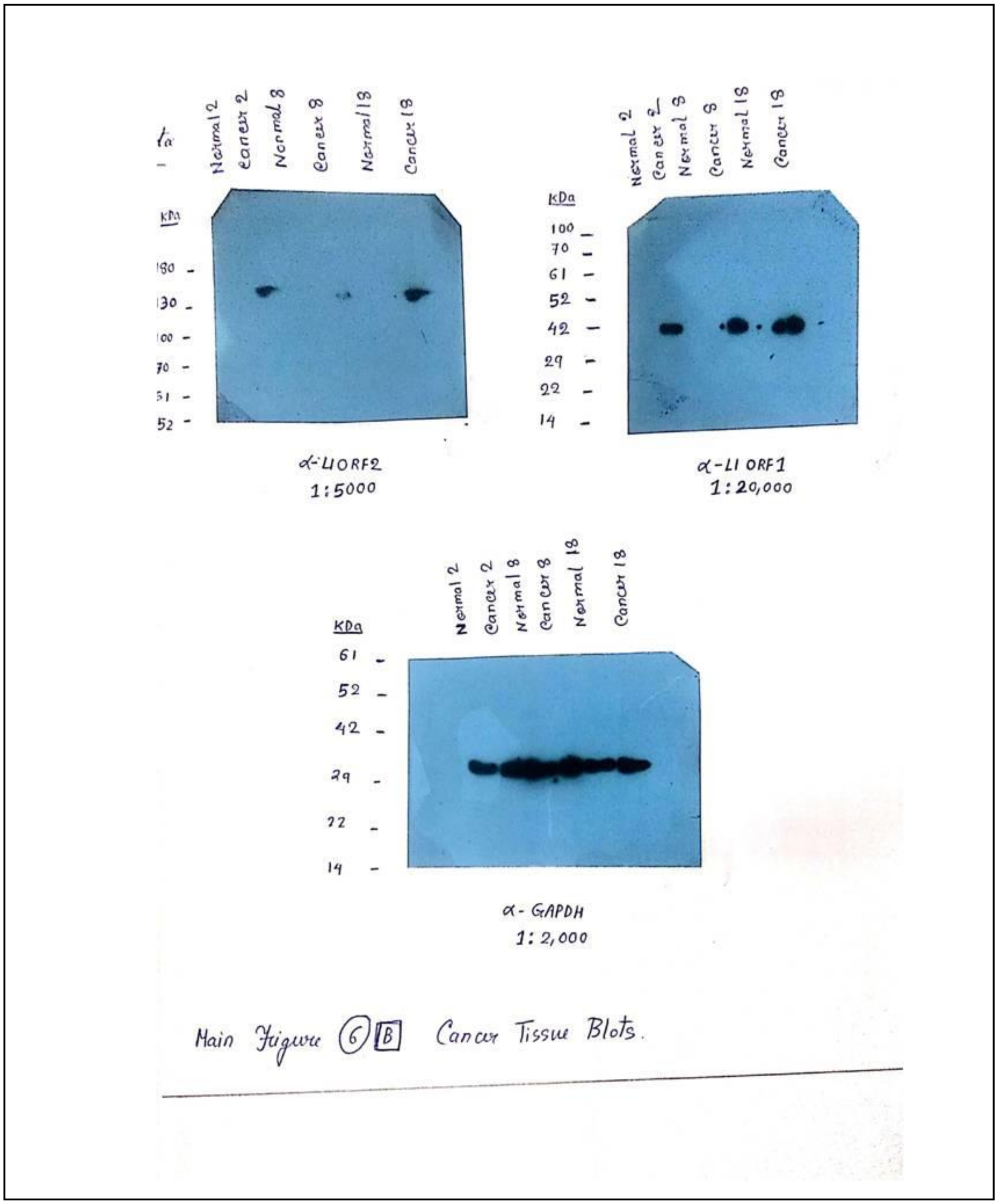
Normal 2
Cancer 2
Normal 8
Cancer 8
Normal 18
Cancer 18
KDa
180
130
100
70
61
52
α-L1ORF2
1:5000
KDa
100
70
61
52
42
29
22
14
α-L1 ORF1
1:20,000
KDa
61
52
42
29
22
14
α-GAPDH
1:2,000
Main Figure 6 B Cancer Tissue Blots.

Page 4

Original Hand written hard copies of Immunoblots shown in supplementary figures attached :

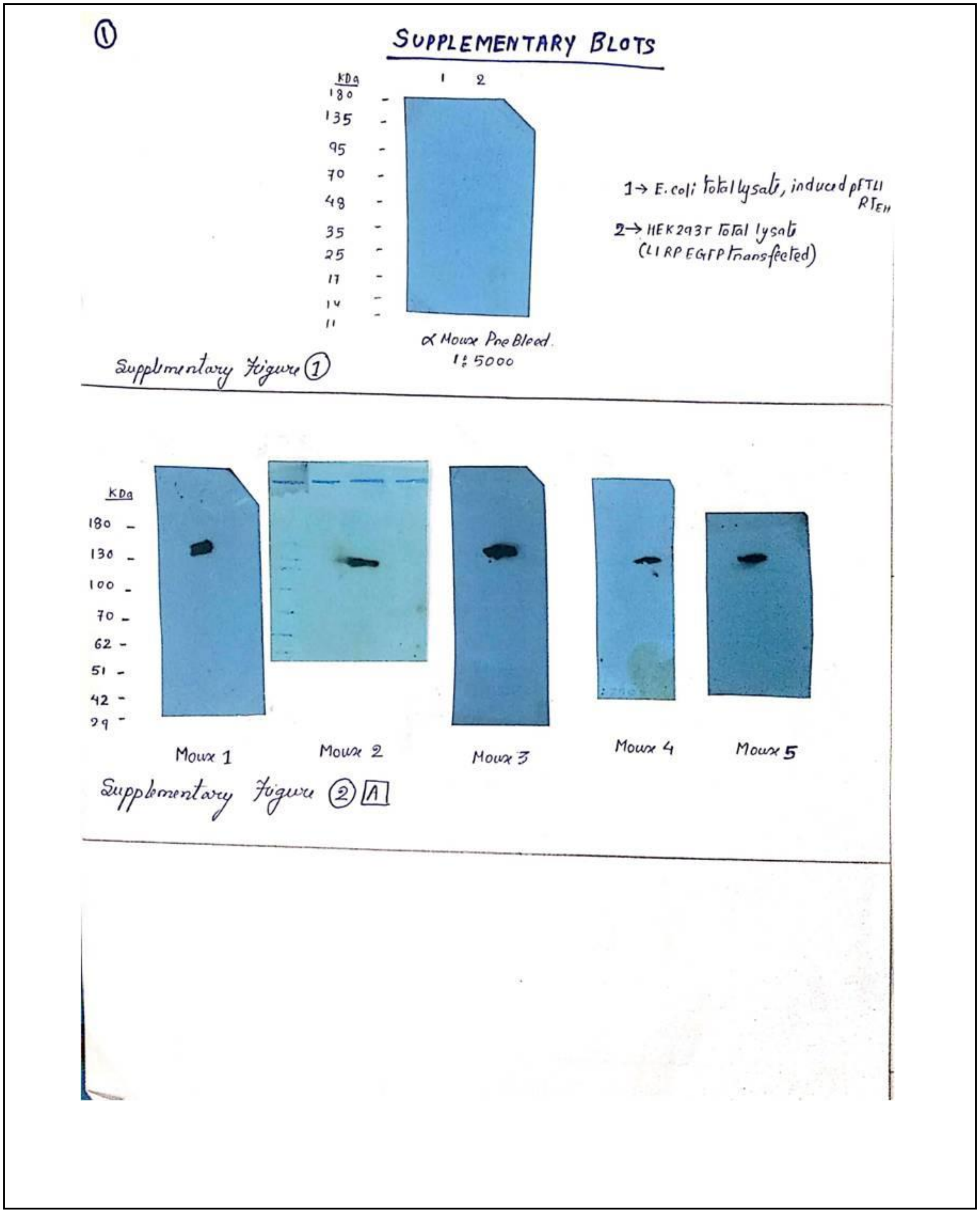

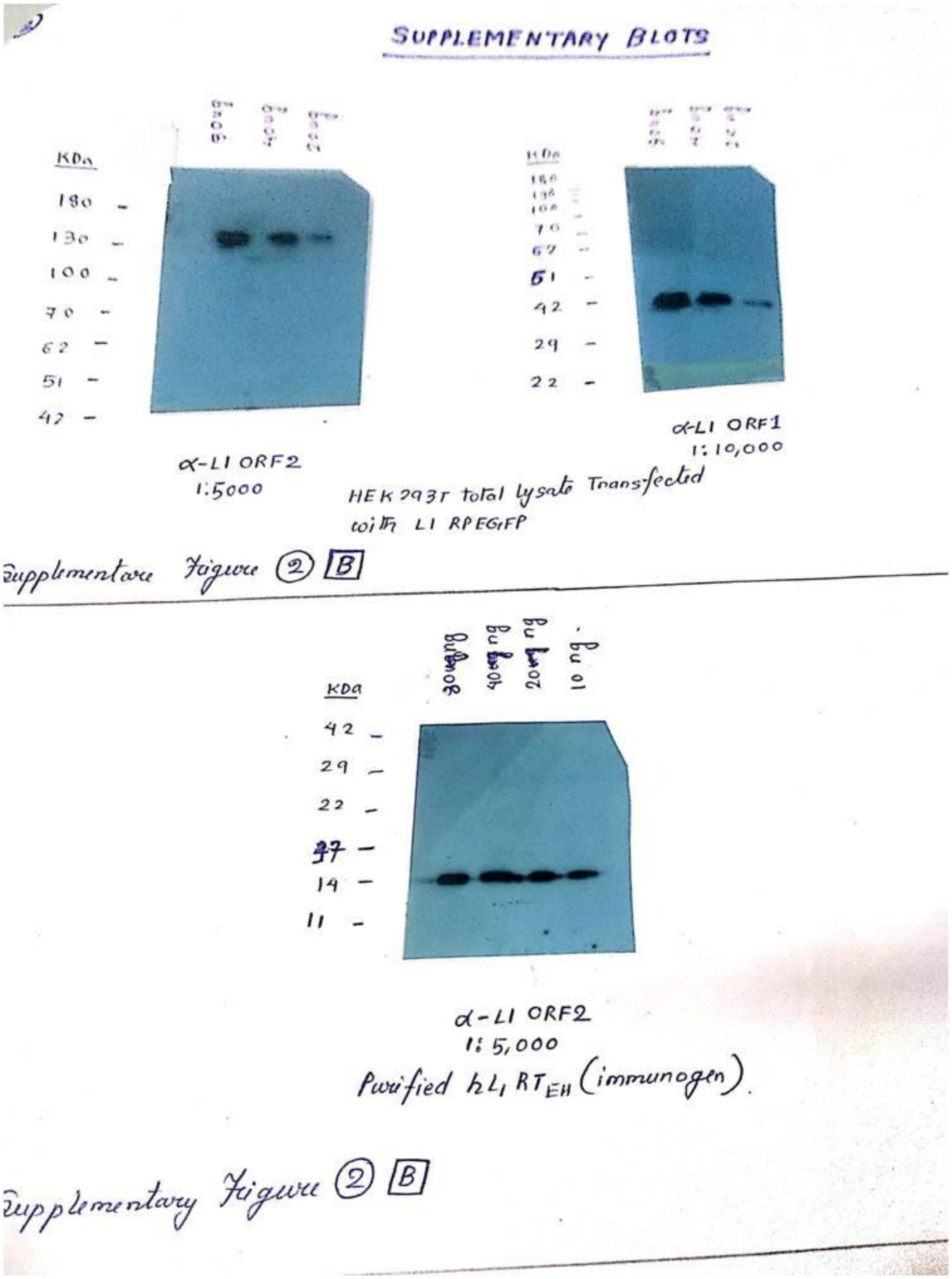
SUPPLEMENTARY BLOTS
KDa
180
130
100
70
62
51
42
α-L1 ORF2
1:5000
KDa
42
29
22
α-L1 ORF1
1:10,000
HEK 293T total lysate Transfected
with L1 RPEGFP
Supplementary Figure ② B
KDa
42
29
22
14
11
α-L1 ORF2
1:5,000
Purified hL1 RT EH (immunogen).
Supplementary Figure ② B

Page 2
